# Elucidating Manganese Single‐Atom Doping: Strategies for Fluorescence Enhancement in Water‐Soluble Red‐Emitting Carbon Dots and Applications for FL/MR Dual Mode Imaging

**DOI:** 10.1002/advs.202414895

**Published:** 2025-01-02

**Authors:** Fucheng Gao, Qiang Fu, Ying Ruan, Can Li, Yandong Wang, Hui Li, Jichao Li, Yanyan Jiang

**Affiliations:** ^1^ Key Laboratory for Liquid‐Solid Structural Evolution and Processing of Materials Ministry of Education Shandong University Jinan 250061 China; ^2^ Department of Urology Shandong Provincial Hospital Affiliated to Shandong First Medical University Jinan 250100 China; ^3^ Key Laboratory of Urinary Diseases in Universities of Shandong (Shandong First Medical University) Jinan 250100 China; ^4^ MOE Key Laboratory of Materials Physics and Chemistry Under Extraordinary Conditions School of Physical Science and Technology Northwestern Polytechnical University Xian 710072 China; ^5^ School of Physics Shandong University Jinan 250100 China

**Keywords:** atomically dispersed manganese atoms, bioimaging, carbon dots, fluorescence enhancement, high photoluminescence quantum yield

## Abstract

The absence of the enhancement of fluorescence in carbon dots (CDs) through doping with transition metal atoms (TMAs) hinders the advancement of multi‐modal bio‐imaging CDs with high photoluminescence quantum yield (PLQY). Herein, Mn‐atomically‐doped R‐CDs (R‐Mn‐CDs) with a high PLQY of 41.3% in water is presented, enabling efficient in vivo dual‐mode fluorescence/magnetic resonance (MR) imaging. The comprehensive characterizations reveal that the incorporation of Mn atoms leads to a Mn‐N_2_O_2_ coordinating structure, resulting in five significant effects: an increase in sp^2^ conjugation domains, a reduction in band gap, a decreased oxidation level, an increase in photo‐excited electron numbers, and the suppression of non‐radiative electron relaxation pathways. Collectively, these factors contribute to the remarkable PLQY of R‐Mn‐CDs. Additionally, the doping of Mn atoms also endows R‐Mn‐CDs with superior MR imaging capabilities due to, which highlights their promising prospect as a dual‐modal bio‐imaging platform for fluorescence/MR imaging. Furthermore, the findings indicate that the introduction of various TMAs, such as Mn, Zn, Ni, and Cu, can universally improve the PLQY of water‐soluble CDs through the construction of TMAs─O bonds. This research provides valuable theoretical insights into the mechanisms underlying the fluorescence enhancement induced by TMAs doping and offers guidance for the future design of high PLQY CDs.

## Graphical abstract

The PLQY of R‐CDs is enhanced by introducing Mn‐N_2_O_2_ binding sites. The formation of Mn─O bonds results in multiple crucial effects: an expansion of sp^2^ conjugation domains, a reduction in bandgap, a lower oxidation level, an increase in photo‐excited electron density, and the suppression of non‐radiative relaxation pathways. Together, these synergistic effects underpin the exceptional PLQY observed in R‐Mn‐CDs.

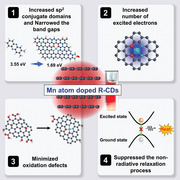

## 1 Introduction

Red light‐emitting carbon dots (R‐CDs, typically with an emission wavelength longer than 590 nm) with high photoluminescence quantum yield (PLQY) have gained significant attention due to their exceptional tissue penetration capabilities, making them promising candidates for in vivo bio‐imaging.^[^ ^1^, ^2^ ^]^ R‐CDs with high PLQY can improve imaging quality, reduce required dosage, and minimize biotoxicity. Recently, several R‐CDs systems have exhibited high PLQY in organic solvents. For example, Yang et al. reported that R‐CDs derived from taxus leaves with a PLQY of 59% in dimethyl sulfoxide,^[^ ^3^ ^]^ while Xiong et al. synthesized R‐CDs from mulberry leaves with a PLQY of 73% in dichloromethane.^[^ ^4^ ^]^ Despite the advancements, these R‐CDs exhibit inadequate hydrophilicity, rendering them susceptible to aggregation and precipitation upon being introduced into the aqueous environments of biological matrices.^[^ ^5^ ^]^ The aggregation of these CDs can lead to fluorescence quenching and may also impose an increased metabolic burden on organs or trigger inflammatory responses, potentially disrupting normal physiological functions.^[^ ^6^, ^7^ ^]^ Therefore, it is crucial to develop R‐CDs that possess both high PLQY and superior water solubility.

The PLQY of R‐CDs in aqueous solution is consistently significantly lower. This phenomenon can be primarily attributed to two factors. First, the interaction between R‐CDs and water involves a protonation‐deprotonation process that results in energy dissipation.^[^ ^8^ ^]^ Second, achieving an optimal balance between the size of sp^2^ domains^[^ ^9^ ^]^ and the oxidation degree^[^ ^10^, ^11^ ^]^ remains a significant challenge. Larger sp^2^ domains can lead to significantly redshifted fluorescence emission but tend to reduce water solubility. Increasing the oxidation degree typically narrows the band gap and enhances solubility, however, this also introduces non‐radiative pathways that ultimately decrease PLQY.^[^ ^12^ ^]^ To address these issues, bovine serum albumin,^[^ ^13^ ^]^ polyetherimide,^[^ ^14^ ^]^ polyethylene glycol,^[^ ^15^ ^]^ and SiO_2_^[^ ^16^ ^]^ are usually utilized to shield R‐CDs to enhance their PLQY by preventing interactions between water molecules and CDs. Although passivating the surface of R‐CDs with hydrophilic molecules, and polymers, as well as creating core‐shell structures, has proven to be an effective approach, there are still limitations in improving the PLQY of CDs in aqueous solutions. Therefore, novel approaches must be developed to obtain R‐CDs with high PLQY in aqueous solution through the rationale regulation of the graphite domain size and oxidation degree.

Doping with transition‐metal atoms (TMAs) has emerged as an effective strategy for modifying the electronic structure and physicochemical properties of CDs.^[^ ^17^, ^18^ ^]^ Research has shown that the incorporation of specific TMAs, such as Mn,^[^ ^19^, ^20^ ^]^ Ni,^[^ ^21^ ^]^ and Fe,^[^ ^22^ ^]^ can significantly improve the PLQY of CDs. However, the mechanisms underlying the enhancement of fluorescence by TMAs doping have yet to be explored. Typically, TMAs chelates are employed as precursors to prepare TMAs‐doped CDs, with N and O atoms as electron donors, creating TMAs‐N or TMAs‐O configurations to reduce oxidation degree. Our recent work elaborated that TMAs doping can decrease the surface functional group content and enlarge graphite domains in CDs. Meanwhile, a reasonable choice of carbon and metal precursors allows for the design of TMAs‐doped CDs with specific binding sites.^[^ ^23^ ^]^ Therefore, the incorporation of TMAs to regulate the sp^2^ conjugated domains and oxidation degree of R‐CDs, along with the establishment of TMA─O bonds through rational selection of carbon and metal precursors, represents an effective strategy for PLQY enhancement.

In this study, we synthesized atomically dispersed Mn‐doped R‐CDs (R‐Mn‐CDs) with a Mn‐N_2_O_2_ configuration. O‐phenylenediamine (OPD), phytic acid (PA), and MnCl_2_ were used as raw materials. The PLQY of R‐Mn‐CDs in water reached 41.3%, which was 3.65 times higher than that of pure R‐CDs. Experimental results showed that Mn atom doping caused an increase in the carbon core size, a reduction in oxidation degree, a narrowing of band gaps, an enhancement in the number of excited electrons, and a suppression of nonradiative relaxation processes. The synergy of these factors collectively contributed to the enhancement of PLQY. Furthermore, theoretical calculations revealed the electron transfer from Mn atoms to N and O atoms, as well as the variation of the electronic structure at Mn‐N_2_O_2_ sites of R‐Mn‐CDs with the size and oxidation degree of the carbon core. The in vivo imaging studies demonstrated the effective performance of R‐Mn‐CDs as red fluorescent probes, exhibiting exceptional biocompatibility and rapid clearance. Mn atom doping also endowed R‐Mn‐CDs with superior *T*_1_‐weighted magnetic resonance (MR) imaging capabilities, highlighting their potential for dual‐mode fluorescence/MR imaging. Furthermore, we also prepared a series of CDs with TMAs‐O (TMAs = Zn, Mn, Cu, Ni) binding sites to demonstrate that the method of constructing TMAs─O bonds for enhancing the PLQY of water‐soluble CDs ought to be universal and effective. The present study elucidates the mechanism underlying Mn atom doping to enhance the fluorescence emission of CDs, and develops a novel strategy for designing water‐soluble CDs with high PLQY.

## 2 Results and Discussion

**Figure 1:**
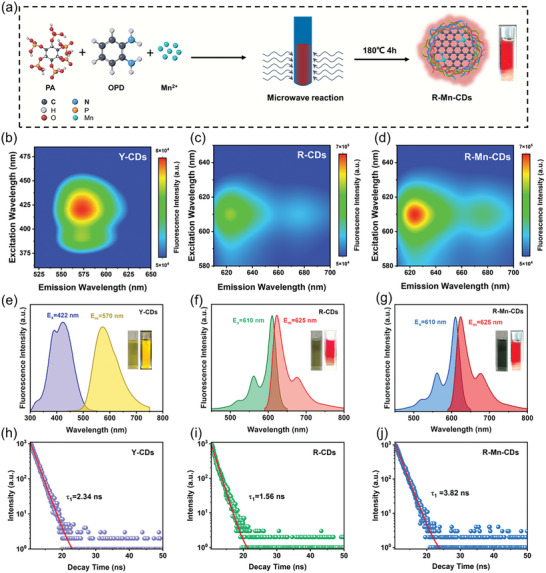
a) Schematic representation of R‐Mn‐CDs preparation. The excitation‐emission mapping of (b) Y‐CDs, c) R‐CDs, and d) R‐Mn‐CDs. Optimal fluorescence excitation, and emission spectra of (e) Y‐CDs, f) R‐CDs, and g) R‐Mn‐CDs. The excitation wavelength is abbreviated as E_x_ and the emission wavelength is abbreviated as E_m_. Photoluminescence decay curves of (h)Y‐CDs, i) R‐CDs, and j) R‐Mn‐CDs.

**Figure** **1**a illustrates the preparation process in this study. We selected OPD as the carbon source, MnCl_2_ as the metal source, and PA as the electron donor due to its abundant phosphate groups that could effectively bond Mn atoms through Mn─O bonds. Additionally, PA established an acidic and oxidative environment that facilitated the polymerization and crosslinking of OPD, which was essential for generating R‐CDs. During the optimization process, we first varied the amounts of OPD and PA. As we increased the amount of PA, we observed a gradual shift in fluorescence emission from yellow to red, accompanied by a continuous enhancement in fluorescence intensity (Figure S1, Supporting Information). To achieve optimal fluorescence and maximal Mn anchoring, we determined that 1 mmol of PA was the ideal quantity when the dosage of OPD was at 0.46 mmol. Subsequently, we focused on fine‐tuning the Mn addition at a PA dosage of 1 mmol. The fluorescence intensity of R‐Mn‐CDs initially increased with rising Mn content but subsequently showed a decline upon the introduction of an excessive amount of Mn. This decrease was attributed to an overproduction of oxides under high‐temperature and high‐pressure reaction conditions, which adversely affected CDs formation and diminished their fluorescence intensity. Ultimately, R‐Mn‐CDs exhibited peak fluorescence emission intensity at 0.5 mmol of Mn (Figure S2, Supporting Information). It is important to highlight that the attempts to synthesize CDs using only PA or its complex with Mn^2+^ ions were unsuccessful (Figure S3, Supporting Information). It could be attributed to the overall functionality of the reaction system, which was restricted to 1. Consequently, the inclusion of OPD, PA, and MnCl_2_ is essential in the successful preparation of R‐Mn‐CDs.

The yellow‐emitting carbon dots (Y‐CDs) synthesized from OPD, as well as the R‐CDs prepared using optimized OPD and PA dosages and R‐Mn‐CDs were selected to investigate the effect of the effects sp^2^ domain, oxidation degree, and Mn atom doping on the CDs. Upon laser excitation at wavelengths between 375—475 nm, Y‐CDs predominantly emitted yellow light ≈550—600 nm (Figure 1b). Further analysis determined that the optimal excitation and emission wavelength for Y‐CDs were 422 and 570 nm, respectively (Figure 1e). The incorporation of PA resulted in enhanced oxidation and polymerization of OPD under highly acidic conditions, leading to R‐CDs displaying intense red‐light emission with the 620—680 nm range when excited at 580—640 nm (Figure 1c). The fluorescence emission spectra revealed a distinct emission peak at 625 nm for R‐CDs, with a shoulder at 675 nm (Figure 1f). Similarly, R‐Mn‐CDs demonstrated comparable fluorescence emission behavior to R‐CDs (Figures 1d,g), yet their optimal emission intensity at 625 nm (under 610 nm excitation) was remarkably enhanced by 14.5 times compared to that of R‐CDs. Their PLQY in water was determined to be 5.8%, 11.3%, and 41.3% for Y‐CDs, R‐CDs, and R‐Mn‐CDs (Figure S4, Supporting Information), respectively. Compared with Y‐CDs, the improvement in the PLQY of R‐CDs was attributed to the doping of P and N atoms, which changed the energy level structure of the luminescence center and affected the characteristics of different vibrational transitions.^[^ ^24^ ^]^ Meanwhile, Mn atom doping also significantly enhanced the PLQY in water. Notably, the PLQY of R‐Mn‐CDs is commendable compared to previously reported water‐soluble R‐CDs (Table S1, Supporting Information). The emission centers of these CDs remained constant as the excitation wavelengths increased from 550 to 620 nm (Figure S5, Supporting Information), indicating excitation‐independent characteristics. The fluorescence lifetime refers to the average duration that the excited‐state electrons require to return to the ground state by the radiation relaxation pathway. In contrast, non‐radiation relaxation pathways describe the mechanisms by which excited‐state electrons revert to the ground state via non‐radiative means, typically leading to a significant reduction in fluorescence lifetime. The average fluorescence lifetimes at the optimal emission wavelength were calculated to be 2.34, 1.56, and 3.82 ns for Y‐CDs, R‐CDs, and R‐Mn‐CDs, respectively, under 475 nm excitation (Figures 1h–j). R‐CDs exhibited the shortest fluorescence lifetimes, potentially attributed to the incorporation of oxidized surface defects that created new relaxation pathways for excited electrons.^[^ ^10^ ^]^ R‐Mn‐CDs demonstrated the longest fluorescence lifetimes in the aqueous phase. This observation suggested that R‐Mn‐CDs possess weaker non‐radiation relaxation pathways.

**Figure 2:**
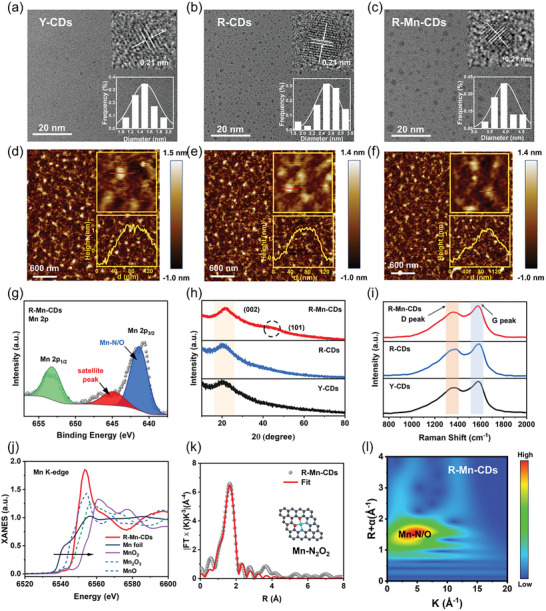
TEM images, HR‐TEM images, and histograms of the size distribution of (a) Y‐CDs, b) R‐CDs, and c) R‐Mn‐CDs. AFM images of (d)Y‐CDs, e) R‐CDs, and f) R‐Mn‐CDs. g) High‐resolution Mn 2p spectra of R‐Mn‐CDs. h) X‐ray diffraction patterns of Y‐CDs (black line), R‐CDs (blue line), and R‐Mn‐CDs (red line). i) Raman spectrum of Y‐CDs (black line), R‐CDs (blue line), and R‐Mn‐CDs (red line). j) XANES spectra at Mn K‐edge of R‐Mn‐CDs and reference samples. k) FT‐EXAFS at the Mn K‐edge of R‐Mn‐CDs and reference samples. l) WT image at the Mn K‐edge of R‐Mn‐CDs.

Transmission electron microscopy (TEM) images showed that the average sizes of Y‐CDs, R‐CDs, and R‐Mn‐CDs were 1.5, 2.4, and 3.6 nm, respectively (**Figures** **2**a–c). The introduction of PA during the synthesis of R‐CDs promoted the polymerization of OPD, additionally, the integration of Mn atoms further contributed to an increase in the dimensions of R‐Mn‐CDs. The lattice‐fringe spacing of these CDs was measured at 0.21 nm, which corresponds to the (100) facet of graphitic carbon.^[^ ^25^ ^]^ The small bright spots (marked with yellow circles) in the high‐angle annular dark‐field scanning transmission electron microscopy (HAADF‐STEM) images of R‐Mn‐CDs in Figure S6 (Supporting Information) suggested enriched single Mn atoms. The atomic force microscopy (AFM) images (Figures 2d–f) revealed distinct topographical heights for Y‐CDs, R‐CDs, and R‐Mn‐CDs at 1.5, 1.8, and 1.4 nm, respectively, suggesting that the strong acidic reaction conditions and the doping of Mn atoms had negligible impact on the overall thickness of CDs. Meanwhile, the core‐level Mn 2p spectrum of R‐Mn‐CDs, shown in Figure 2g, displayed a peak at 645.1 eV corresponding to a satellite peak. The peaks at 641.4 and 653.3 eV for Mn 2p_3/2_ and Mn 2p_1/2_, respectively, indicated a +2 valence state associated with the presence of Mn─N/O bonds,^[^ ^26^, ^27^ ^]^ further supporting the existence of Mn atoms. The X‐ray diffraction patterns (Figure 2h) consistently exhibited peaks at 21.4°, which are attributed to the (001) crystalline facet. Meanwhile, R‐Mn‐CDs displayed an additional peak at 45.3°, corresponding to the (101) crystalline facet of graphitic carbon,^[^ ^28^ ^]^ indicating a more graphene‐like carbon core. The Raman spectra for Y‐CDs, R‐CDs, and R‐Mn‐CDs, as shown in Figure 2i, revealed two prominent peaks at 1350 and 1580 cm^−1^, indicative of the D‐ and G‐bands, respectively. The *I*_D_/*I*_G_ ratios for Y‐CDs and R‐CDs were measured at 1.16 and 1.32, respectively, indicating a higher content of sp^3^ carbon in R‐CDs. Despite being larger than Y‐CDs, the effective conjugation with the carbon core in R‐CDs appeared to be limited.^[^ ^29^ ^]^ In contrast, R‐Mn‐CDs exhibited a lower *I*_D_/*I*_G_ ratio of 0.83, signifying an increased proportion of sp^2^ carbon domains. The incorporation of Mn atoms facilitated the expansion of these sp^2^ domains in R‐Mn‐CDs, thereby enhancing the conjugation with the carbon cores.

The X‐ray absorption near‐edge structure (XANES) and Fourier‐transformed extended X‐ray absorption fine structure spectra (FT‐EXAFS) were recorded to determine· the precise binding sites of Mn atoms on R‐Mn‐CDs. The Mn K‐edge spectrum revealed that the absorption thresholds of R‐Mn‐CDs were positioned between MnO and Mn_2_O_3_ (Figure 2j), suggesting an intermediate oxidation state for Mn atoms falling between Mn^2+^ and Mn^3+^. The EXAFS curves presented in Figure S7 (Supporting Information) revealed a major peak at 1.67 Å, attributed to backscattering interactions between Mn, O, and N atoms. Notably, unlike the spectra for Mn foil and MnO, no signals for the Mn‐Mn scattering path at 2.27 and 2.70 Å were detected indicating a uniform distribution of single Mn atoms in R‐Mn‐CDs.^[^ ^30^ ^]^ Further analysis through EXAFS curve fitting in both R‐ and k‐spaces confirmed that the coordination number for the Mn site was represented as Mn‐N₂O₂ (Figure 2k). The average bond lengths for Mn─N and Mn─O were determined to be 2.00 and 2.19 Å, respectively (Table S2, Supporting Information). The backscattering atoms were identified through wavelet transform (WT) of Mn K‐edge EXAFS oscillations. The WT contour plots for R‐Mn‐CDs compared with those of Mn foil, MnO₂, Mn₂O₃, and MnO (Figure S8, Supporting Information) displayed a prominent feature in both R‐space (≈1.59 Å) and k‐space (≈4.2 Å) (Figure 2l), indicative of the presence of Mn─N/O bonding without any detectable signals related to Mn–Mn interactions. These results further affirmed that Mn atoms were atomically dispersed in R‐Mn‐CDs. The observed coordination structure of Mn‐N₂O₂ deviates from the original Mn‐O_6_ structure where Mn was coordinated with PA. This change suggested a replacement of O ligands by N during hydrothermal synthesis. This phenomenon may be attributed to the decomposition of PA in a hydrothermal process, resulting in the disruption of Mn─O bonds between the phosphorus carboxyl group, hydroxyl group, and Mn^2+^ ions. Furthermore, the aromatization process involved in the formation of CDs prompted the shift in the coordination sphere of Mn atoms from O to N, consistent with prior reports.^[^ ^23^ ^]^ These results validated the existence of individual Mn atoms in R‐Mn‐CDs, with Mn‐N_2_O_2_ binding structure.

**Figure 3:**
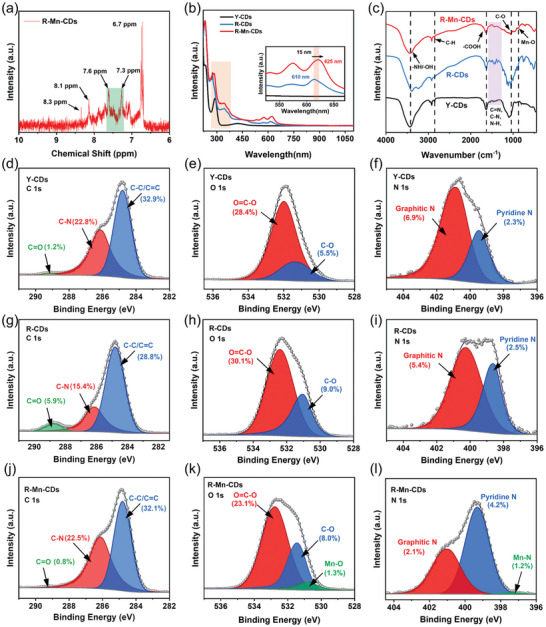
a) ^1^H NMR spectrum of R‐Mn‐CDs. b) UV–vis spectra of Y‐CDs (black line), R‐CDs (blue line), and R‐Mn‐CDs (red line). c) FTIR spectra of Y‐CDs (black line), R‐CDs (blue line), and R‐Mn‐CDs (red line). Core‐level XPS spectra of (d) C 1s, e) O 1s, and f) N 1s orbitals of Y‐CDs. Core‐level XPS spectra of (g) C 1s, h) O 1s, and i) N 1s orbitals of R‐CDs. The core‐level XPS spectra of (j) C 1s, k) O 1s, and l) N 1s orbitals of R‐Mn‐CDs.

Additionally, we conducted a comprehensive analysis of the structural composition, photo‐absorption behavior, and surface moieties, to investigate the mechanism underlying the enhanced PLQY upon Mn atoms doping. As shown in the proton nuclear magnetic resonance (NMR) spectroscopy of R‐Mn‐CDs (**Figure** **3**a), the chemical shift observed at 6.6 ppm indicated the presence of C─H bonds with the aromatic ring, while shifts at 7.3 and 7.6 ppm were associated with phenyl‐C structures located distantly from the amino group.^[^ ^31^ ^]^ Additionally, peaks at 8.1 and 8.3 ppm corresponded to aromatic structures on the armchair side,^[^ ^32^ ^]^ collectively suggesting that polycyclic aromatic hydrocarbon structures were present in R‐Mn‐CDs.^[^ ^33^ ^]^ The UV–vis spectra further elucidated the light absorption properties of Y‐CDs, R‐CDs, and R‐Mn‐CDs at identical concentrations. As shown in Figure 3b, all three groups displayed a prominent absorption band ≈300 nm, which is attributed to the π–π^*^ transition of C═C in conjugated aromatic structure.^[^ ^34^ ^]^ The Y‐CDs displayed an absorption peak at 425 nm linked to n‐π^*^ transitions involving C═N structures from pyridine‐N in the carbon core.^[^ ^35^ ^]^ In contrast, R‐CDs and R‐Mn‐CDs showed red‐shifts in their n‐π^*^ transitions for C═N and C─N─C bonds toward the range of 600—650 nm, indicating that these two types possess lower bandgaps compared to Y‐CDs. Notably, an enhanced absorbance in R‐Mn‐CDs indicated that Mn atom doping extended the conjugated sp^2^ domain in carbon cores. Tauc plot analysis confirmed this observation by revealing band gaps of 2.09 eV for Y‐CDs, 1.68 eV for R‐CDs, and a reduced value of 1.44 eV for R‐Mn‐CDs (Figure S9, Supporting Information). It is essential to emphasize that the fluorescence of R‐CDs, prepared by OPD, originates from a molecular state.^[^ ^24^, ^31^, ^36^ ^]^ Thus, despite a slight reduction in bandgap due to Mn doping, there was no corresponding redshift observed in the fluorescence emission spectrum of R‐Mn‐CDs.

Fourier transform infrared (FTIR) spectroscopy was further utilized to analyze the surface functional groups of these CDs. As illustrated in Figure 3c, these CDs displayed comparable absorption bands at ≈3450 and 2850 cm^−1^, which are indicative of the ─OH/─NH and C─H stretching vibrations, respectively. The presence of polyaromatic structures in these CDs was further confirmed by additional bands observed at ≈1524, 1642, and 1427 cm^−1^, which could be attributed to the C═C, C─N, and N─H stretching vibrations, respectively.^[^ ^37^ ^]^ Notably, a band at 1048 cm^−1^ associated with C─O stretching was exclusively detected in R‐CDs and R‐Mn‐CDs synthesized under acidic conditions. R‐CDs exhibited a pronounced band at 1727 cm^−1^ that signifies ─COOH stretching vibration, reflecting a higher degree of oxidation. R‐Mn‐CDs revealed an additional band at 883 cm^−1^ linked to Mn─N/O stretching vibrations,^[^ ^19^ ^]^ consistent with findings from XANES results. The reduced peak intensities for both C─O and ─COOH in R‐Mn‐CDs suggested that the degree of oxidation was partially offset by the formation of Mn─O bonds.

The X‐ray photoelectron spectroscopy (XPS) survey scan demonstrated the composition variation from Y‐CDs to R‐Mn‐CDs. As shown in Figure S10 (Supporting Information), Y‐CDs comprised 56.9% C, 33.9% O, and 9.2% N. In contrast, R‐CDs contained 50.1% C, 39.1% O, 7.9% N, and an additional 2.9% P. The composition of R‐Mn‐CDs was found to consist of C (55.4%), O (32.4%), N (7.5%), and P (3.1%). Additionally, the Mn content was determined to be 1.6% by XPS, aligning closely with the value of 1.46% obtained through inductively coupled plasma spectroscopy (ICP) (Table S3, Supporting Information). The high‐resolution C 1s spectra of all the CDs could be deconvoluted into three distinct peaks representing C─C/C═C (284.8 eV), C─N (286.1 eV), and C═O (289.2 eV).^[^ ^38^ ^]^ The introduction of PA led to a decrease in both the C─C/C═C and C─N content while increasing the proportion of C═O groups in R‐CDs compared to Y‐CDs (Figures 3d,g). This change indicated that R‐CDs were more oxidized than Y‐CDs, which was further supported by the increased presence of C─O and O═C─O groups observed in the high‐resolution O 1s spectra for R‐CDs (Figures 3e,h). However, doping with Mn atoms led to an increase in the content of sp^2^‐C represented by C─C/C═C while reducing sp^3^‐C indicated by decreased levels of C═O in R‐Mn‐CDs (Figure 3j). Meanwhile, the O 1s spectra of R‐Mn‐CDs affirmed the presence of the Mn─O bond alongside a reduction in oxygen‐containing functional groups (Figure 3k). This observation was corroborated by electron paramagnetic resonance (EPR) spectrum analysis presented in Figure S11 (Supporting Information). Additionally, the high‐resolution N 1s spectra across all CDs (Figures 3f,i,l) demonstrated that the N species were predominantly present as pyridine N and graphitic N.^[^ ^30^ ^]^ The equivalent presence of Mn─N and Mn─O bonds further validated the bonding structure consistent with Mn‐N_2_O_2_ configurations identified through XANES findings. As a consequence, the presence of Mn atoms reduced band gaps, expanded the conjugated network of R‐Mn‐CDs, and partially mitigated the oxidation degree caused by the harsh acidic reaction conditions in R‐Mn‐CDs.

**Figure 4:**
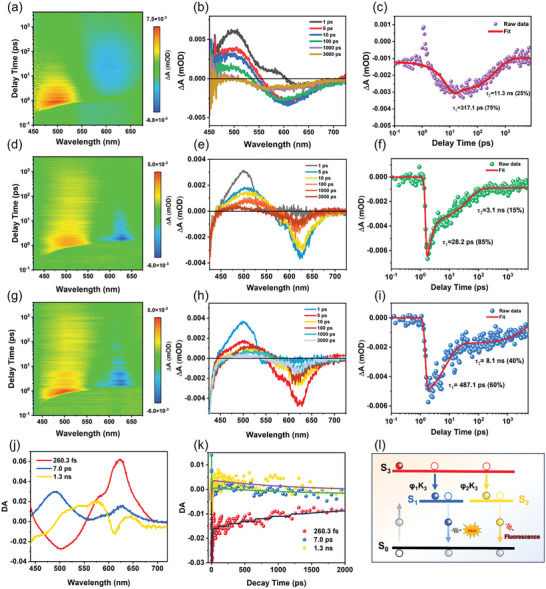
a) 2D pseudocolor map of TA spectra of Y‐CDs. b) TA spectra of Y‐CDs were recorded at a specified delay time under 400 nm excitation. c) TA kinetic traces of Y‐CDs at 625 nm within 7000 ps. d) 2D pseudocolor map of TA spectra of R‐CDs. e) TA spectra of R‐CDs recorded at specified delay time under 400 nm excitation. f) TA kinetic traces of R‐CDs at 625 nm within 7000 ps. g) Pseudo‐3D TA spectra of R‐Mn‐CDs. h) TA spectra of R‐Mn‐CDs were recorded at a specified delay time under a 400 nm excitation. i) TA kinetic traces of R‐Mn‐CDs at 625 nm within 7000 ps. j) Decay associated difference spectra obtained by singular value decomposition (SVD), k) Global fitting results of TA principal component decay kinetic traces, l) Photoluminescence transition model proposed for R‐Mn‐CDs.

Femtosecond transient absorption (TA) measurement was further performed utilizing a 400 nm pulsed laser with an intensity of 0.21 mW cm^−2^ to investigate the PL mechanism of these CDs.^[^ ^39^ ^]^ **Figures** **4**a,d,g presented the 2D pseudocolor map of TA spectra across a probe wavelength range of 425—675 nm with scan delay times extending from −1 to 7000 ps. The observed photophysical processes across all CD samples displayed similar characteristics. Notably, negative signals (blue) with the wavelength range of 600—650 nm indicated ground state bleaching (GSB), while positive signals (yellow and red) in the range of 450—550 nm signified excited state absorption (ESA).^[^ ^40^ ^]^ The ESA signal of R‐Mn‐CDs was significantly stronger than that of R‐CDs, suggesting enhanced charge generation attributed to Mn doping.^[^ ^41^ ^]^ Further analysis of TA spectra at different delay times (Figures 4b,e,h) revealed that the ESA signal for Y‐CDs, R‐CDs, and R‐Mn‐CDs peaked at ≈1 ps before gradually decaying over time. The dynamics of photogenerated charges were monitored at a wavelength of 625 nm (Figures 4c,f,i). All samples exhibited decay curves that could be fitted with a bi‐exponential function comprising rapid decay component τ₁ and slow component τ₂ associated with non‐radiative and radiative recombination processes, respectively. Specifically, Y‐CDs demonstrated decay constants of τ₁ = 317.1 ps and τ₂ = 11.3 ns. In contrast, R‐CDs showed significantly shorter lifetimes with τ₁ = 28.2 ps and τ₂ = 3.1 ns, ≈11 times shorter than those observed in Y‐CDs, likely due to electron trapping by oxidized surface defects. This was further supported by an increase in the weight of τ₁ for R‐CDs, indicating an additional non‐radiative decay pathway linked to surface oxidation. Conversely, R‐Mn‐CDs exhibited prolonged electron relaxation times with τ_1_ = 487.1 ps and τ_2_ = 8.1 ns, which were 17.3 and 2.6 times greater than those of R‐CDs, respectively. The reduction in the weight of τ₁ for R‐Mn‐CDs suggested that Mn‐O coordination effectively mitigated non‐radiative processes associated with oxidized surface defects.

For R‐Mn‐CDs, three principal components were identified through singular value decomposition (SVD) with significance coefficients of 0.29, 0.19, and 0.05, respectively (Figure 4j). A global fitting analysis further elucidated the decay‐associated difference spectra (DADS), with fitted lifetimes of 260.3 ± 260 fs, 7.0 ± 2.4 ps, and 1.3 ± 0.3 ns (Figure 4k). Based on the photoluminescence mechanisms, a transition model has been proposed (Figure 4l).^[^ ^42^ ^]^ This model includes three excited states: an excited state (S_3_), and two metastable excited states (S_1_ and S_2_). Upon photon absorption, hot carriers are generated in S_3_ and subsequently relax through electron, phonon, and acoustical phonon scattering with 260.3 ± 260 fs. During this relaxation process, the hot carriers exhibit a 60.4% probability of populating the excited state S_1_, while a 39.6% probability is assigned to the excited state S_2_. The carriers in S_1_ undergo non‐radiative decay to return to the ground state (S_0_) with an average time of 7.0 ± 2.4 ps through collisions, while the carriers in S_2_ return to S_0_ radiatively with 1.3 ± 0.3 ns through electron‐hole recombination, emitting photons in the process. These findings underscore that the presence of Mn─O bonds in R‐Mn‐CDs significantly impeded the non‐radiative transition process of the excited state.

**Figure 5:**
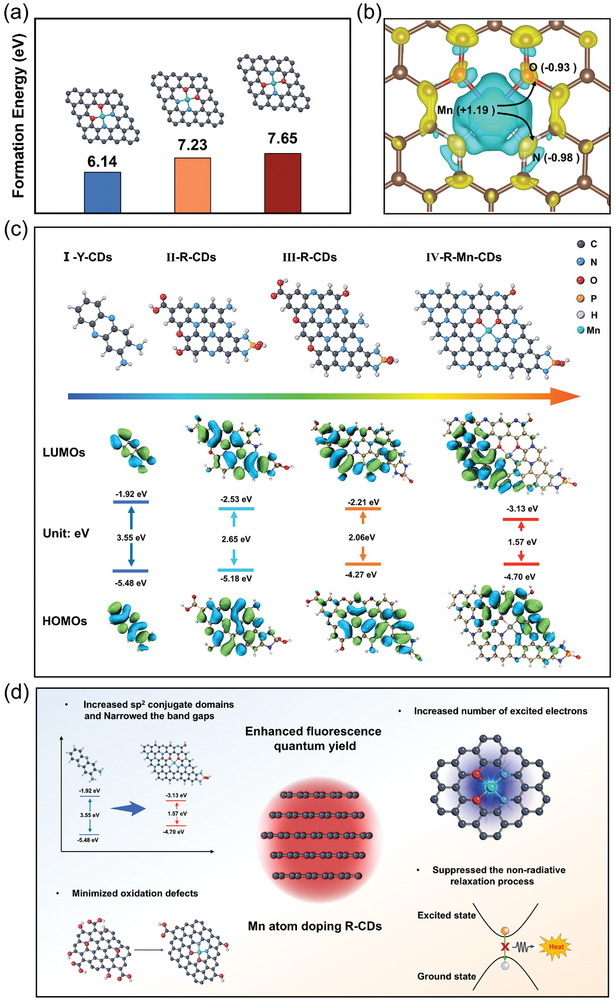
a) Formation energy and structures of three potential configurations of R‐Mn‐CDs. b) Isosurfaces of differential charge density of Mn‐N_2_O_2_ sites. Yellow and blue reflect the accumulation and depletion of charge, respectively. Partial charges on interested atoms are displayed in the brackets. c) Molecular structures of the four models and calculated LUMOs and HOMOs of I‐Y‐CDs, II‐R‐CDs, III‐R‐CDs, and IV‐R‐Mn‐CDs (from left to right). d) The schematic diagram of the mechanism of Mn atom doping to enhance the fluorescence quantum yield of R‐CDs.

We next conducted theoretical investigations to unravel the mechanism underlying Mn atom doping for PLQYs enhancement.^[^ ^43^ ^]^ The Mn atom in R‐Mn‐CDs was found to be in Mn‐N_2_O_2_ coordination configuration, however, the precise arrangement of N and O atoms in this structure remained ambiguous. We proposed three potential configurations: I‐Mn‐N_2_O_2_, II‐Mn‐N_2_O_2_, and III‐Mn‐N_2_O_2_ (from left to right), as depicted in **Figure** **5**a. Among these options, the I‐Mn‐N_2_O_2_ configuration exhibited the lowest formation energy and was selected for subsequent calculations. Furthermore, as shown in Figure 5b, the transfer of charge from Mn atoms to N and O atoms was observed after the incorporation of single‐atom Mn. This charge transfer can be attributed to the higher electronegativity of N and O compared to that of Mn, as indicated by blue regions representing electron loss and yellow regions indicating electron gain. Bader charge analysis quantified this phenomenon: Mn atoms experienced an electron loss corresponding to a partial charge of +1.19 |e|, whereas N and O atoms gained electrons with partial charges of −0.98 |e| and −0.93 |e| respectively.

The comprehensive purification process effectively eliminates the majority of fluorescent molecules, suggesting that the red emitters in CDs were primarily located in the carbon core. Meanwhile, the P element, presenting as P─N and P─O/P═O bonds existed in R‐CDs and R‐Mn‐CDs (Figure S12, Supporting Information). The complex structures of CDs present significant challenges when attempting to create accurate molecular models. Hence, drawing on the polymerization behavior of OPD in acidic conditions,^[^ ^24^ ^]^ the genesis mechanism of TMA‐doped CDs,^[^ ^19^, ^23^ ^]^ and the observed disparities in oxidation degree and sizes across various CDs under practical conditions, we proposed four potential structural models labeled I‐Y‐CDs, II‐R‐CDs, III‐R‐CDs, and IV‐R‐Mn‐CDs to explore how electronic structures relate to size increases and specific oxidation degree (Figure S13, Supporting Information). In these models, oxidizing groups were strategically positioned at the periphery to simplify analysis. We assessed the optimized geometry of these models and determined the highest occupied molecular orbital (HOMO) and lowest unoccupied molecular orbital (LUMO) distributions in vacuum. The calculated band gaps for these models were found to be 3.55 eV for I‐Y‐CDs, 2.65 eV for II‐R‐CDs, 2.06 eV for III‐R‐CDs, and 1.57 eV for IV‐R‐Mn‐CDs (Figure 5c), respectively, revealing a notable reduction in band gap values corresponding with increased oxidation defects and enhanced conjugation segments. The band gap for IV‐R‐Mn‐CDs of 1.57 eV aligns closely with an experimentally determined value of 1.44 eV for R‐Mn‐CDs.

Based on both the experimental and computational results, the possible mechanism through which Mn atoms contribute to the enhanced PLQY of R‐CDs in water is illustrated in Figure 5d. Mn atoms are integrated into the carbon core of R‐Mn‐CDs, forming a Mn‐N₂O₂ structure. This Mn doping induces five key effects: it expands the sp^2^ domains in the R‐Mn‐CDs, partially reduces the degree of oxidation, narrows the band gaps, and increases the population of excited electrons. Moreover, the interaction between Mn and O atoms mitigates the non‐radiative relaxation of excited electrons. Together, these factors synergistically contribute to the significantly high PLQY observed in R‐Mn‐CDs in aqueous environment.

We subsequently assessed the fluorescence emission stability of Y‐CDs, R‐CDs, and R‐Mn‐CDs. Following exposure to different metal salt solutions or a storage period of 30 days, the fluorescence intensity of all three CDs showed negligible variation (Figure S14, Supporting Information). Under continuous illumination at a wavelength of 432 nm (2 W cm^−2^) for yellow Y‐CDs, and at 610 nm (2 W cm^−2^) for R‐CDs and R‐Mn‐CDs over a duration of 60 min, these CDs demonstrated excellent photostability and resistance to photobleaching, maintaining consistent fluorescence intensity (Figure S15, Supporting Information). We also explored the impact of temperature on their fluorescence properties. Across a temperature range from 10 to 90 °C, the fluorescence intensity remained nearly constant (Figure S16a, Supporting Information). However, pH levels significantly influenced their fluorescence behavior. As the pH increased from 2 to 12, there was a gradual decline in fluorescence intensity of Y‐CDs, R‐CDs, and R‐Mn‐CDs, with a complete quenching occurring within the pH range of 10 to 12 (Figure S16b, Supporting Information). This reduction could be attributed to the deprotonation process that facilitated non‐radiative relaxation pathways for excited electrons. Notably, in the physiologically relevant pH range of 4–8, Y‐CDs retained ≈45% of their initial fluorescence intensity, while R‐CDs and R‐Mn‐CDs maintained ≈70% and 50%, respectively. The exceptional fluorescent performance exhibited by R‐Mn‐CDs under the aforementioned conditions suggested their remarkable suitability as imaging probes for both in vivo and in vitro applications.

**Figure 6:**
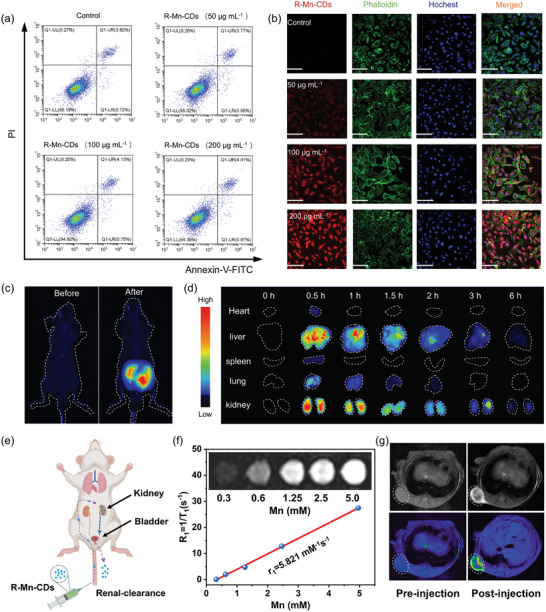
a) Apoptosis results of PC‐3 cells after being incubated with different concentrations of R‐Mn‐CDs (0, 50, 100, and 200 µg mL^−1^) for 72 h. b) Confocal fluorescence confocal image of PC‐3 cells treated with different concentrations of R‐Mn‐CDs. The red light emission is attributed to R‐Mn‐CDs, while the green light is produced by gibberellin peptide. In addition, the blue light originates from DPAI. Scale bar = 80 µm c) In vivo fluorescence images of a mouse before and after intraperitoneal injection of R‐Mn‐CDs aqueous solution (10 mg Kg^−1^). d) In vivo fluorescence images of major organs (heart, liver, spleen, lung, and kidney) in mice after intravenous injection of R‐Mn‐CDs (10 mg Kg^−1^) at different times (0, 0.5, 1, 1.5, 2, 4, and 6 h). These images were captured using the IVIS Spectrum in vivo imaging system from PerkinElmer, with a 580 nm excitation light source and a 625 nm emission signal. e) Illustration for the renal clearance of R‐Mn‐CDs in the mouse. f) In vitro MRI and *T*_1_ relaxation rate (*r*_1_) of R‐Mn‐CDs in buffer solution at different Mn concentrations employed (0.3, 0.6, 1.25, 2.5, and 5.0 mm). g) In vivo MRI imaging, conducted before and after 1.5 h following the direct injection of R‐Mn‐CDs into PC‐3 tumors.

The cytotoxicity of R‐Mn‐CDs was evaluated using the standard CCK‐8 method with PC‐3 cells. There was no observed inhibition of cell viability even at concentrations up to 200 µg mL^−1^ (Figure S17, Supporting Information). After 72 h incubation with different R‐Mn‐CDs (0, 50, 100, and 200 µg mL^−1^), the cell viability remained over 95%, suggesting negligible cytotoxicity (**Figure** **6**a). Moreover, bright intracellular red fluorescence was visible in PC‐3 cells after 4‐h incubation with R‐Mn‐CDs, indicating its potential application in fluorescence bio‐imaging (Figure 6c). Interestingly, the fluorescence with PC‐3 cells decreased after 48 h incubation with 50 µg mL^−1^ R‐Mn‐CDs, possibly because of cell division (Figure S18, Supporting Information). Although R‐Mn‐CDs exhibited excellent performance in cell imaging, their surface negative charge (‐39.4 mV) limited their penetration into the negatively charged cell membrane, thereby reducing the uptake efficiency of R‐Mn‐CDs by PC‐3 cells. To address this issue, we treated R‐Mn‐CDs with L‐arginine (L‐Arg) to impart a positive charge onto them, reversing their surface zeta potential to +30.3 mV (Figure S19, Supporting Information). Due to the positive charge, the uptake of L‐Arg@R‐Mn‐CDs by PC‐3 cells was significantly increased (Figure S20, Supporting Information).

Furthermore, the internalization of R‐Mn‐CDs into PC‐3 cells was investigated by perturbing the endocytosis‐mediated internalization. The cellular‐uptaking process was observed under a confocal fluorescence microscope. To identify the specific endocytic pathways involved, various inhibitors were applied, including chlorpromazine (CPZ, for blocking clathrin‐mediated endocytosis), filipin for caveolae‐mediated uptake, and amiloride for inhibiting micropinocytosis. Inhibitor concentrations were confined to those that did not affect the cell viability (Figure S21, Supporting Information). The results revealed that CPZ exhibited the most obvious inhibitory effect on the uptake of R‐Mn‐CDs, reducing the relative internalization rate to ≈50%. It was indicated that the internalization of R‐Mn‐CDs in PC‐3 cells could be dominated by clathrin‐mediated endocytosis. In addition, treatments with sodium azide (SA), an energy inhibitor, or exposure to low temperatures (4 °C) significantly reduced R‐Mn‐CDs internalization, demonstrating that the endocytosis process was energy‐dependent (Figure S22, Supporting Information).

To evaluate the in vivo fluorescence imaging capability, biocompatibility, and metabolism of R‐Mn‐CDs, we performed intraperitoneal and tail vein injections in nude mice. As shown in Figure 6c, 5 min after intraperitoneal injection, a significant fluorescence signal was observed in the abdominal area, demonstrating the potential of R‐Mn‐CDs for in vivo fluorescence imaging. Following tail vein injection, major organs (heart, lungs, liver, spleen, and kidneys) were collected at various time points (0, 0.5, 1, 1.5, 2, 3, and 6 h) for fluorescence analysis (Figures 6d,e). The changes in fluorescence signals in these organs indicated that the liver, lungs, and kidneys were effectively involved in the excretion of R‐Mn‐CDs within 6 h post‐injection. The metabolism of R‐Mn‐CDs by the liver and lungs is attributed to their tendency to slightly aggregate at high concentrations, forming relatively larger self‐assembled nanoparticles (Figure S23, Supporting Information). These nanoparticles were more likely to be recognized and phagocytized by liver macrophages. Additionally, R‐Mn‐CDs were detected in the urine of mice (Figure S24, Supporting Information), indicating efficient renal clearance and high metabolic stability^[^ ^44^ ^]^ (Figure 6e). Histological examination using H&E staining showed no notable inflammatory response compared to the control group, confirming the excellent biocompatibility (Figure S25, Supporting Information).

Theoretically, Mn^2+^ with unpaired *3d* electrons can be used as an effective *T*_1_‐weighted MR imaging contrast agent.^[^ ^45^, ^46^ ^]^ The *T*_1_‐weighted magnetic resonance signal exhibited a linear increase in the Mn content in R‐Mn‐CDs. The longitudinal relaxation (*r*_1_) of R‐Mn‐CDs was estimated to be 5.82 mm ^−1^ s^−1^(Figure 6f), demonstrating a competitive performance compared to other MR imaging contrast agents (Table S4, Supporting Information). In vivo MR imaging performance of R‐Mn‐CDs was initially evaluated in mice post intravenous injection of R‐Mn‐CDs (10 mg kg^−1^) followed by scanning with an MR imaging system. An enhanced signal was detected in the kidney 1.5 h after injection (Figure S26, Supporting Information). Furthermore, R‐Mn‐CDs were directly injected into the tumors of PC‐3 tumor‐bearing mice. After 1.5 h, distinct imaging signals were observed in the tumor region (Figure 6g). These findings demonstrate the exceptional in vivo fluorescence ‐MR dual‐mode imaging capabilities of R‐Mn‐CDs, particularly in tumor tissues.

To further validate the efficacy of TMAs─O bonds in enhancing the PLQY of CDs, we used EDTA and ethanolamine as carbon sources. MnCl_2_, ZnCl_2_, NiCl_2_, and CuCl_2_ were adopted as metal sources to synthesize Mn‐CDs, Zn‐CDs, Ni‐CDs, and Cu‐CDs, respectively. Pure carbon dots (P‐CDs), synthesized only using EDTA and ethanolamine, served as the control. EDTA and ethanolamine contain abundant oxygen‐containing functional groups that can coordinate with TMAs, ensuring the introduction of TMAs─O bonds. The doping levels of Mn, Zn, Ni, and Cu were quantified using ICP analysis, revealing respective values of 1.32%, 1.61%, 2.15%, and 2.63%. The difference in the doping levels under identical reaction conditions might be associated with the stability of their corresponding coordination compounds EDTA‐TMA. The stability constant (K) serves as the equilibrium constant for complexation reactions, indicating the strength of binding between ligands and solvated ions. The stability of coordination compounds could be assessed by the value of K, with a higher K indicating greater compound stability.^[^ ^47^ ^]^ The comparison of K values for EDTA‐TMA complexes elucidated a stability order: EDTA‐Mn (13.8) < EDTA‐Zn (16.4) < EDTA‐Ni (18.5) < EDTA‐Cu (18.8).^[^ ^48^ ^]^ This suggested a positive correlation between the stability of the carbon precursors and the doping levels of TMAs.

**Figure 7:**
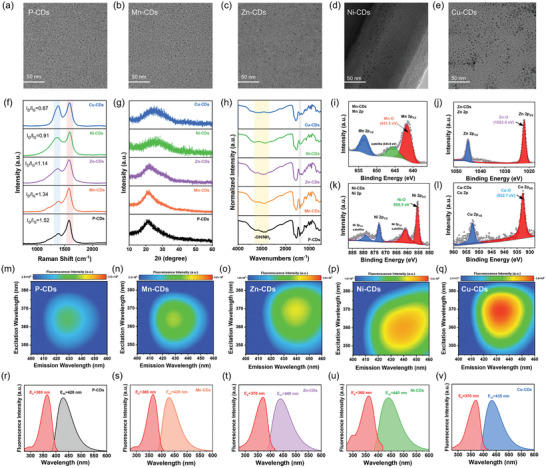
TEM images of (a) P‐CDs, b) Mn‐CDs, c) Zn‐CDs, d) Ni‐CDs and e) Cu‐CDs. f) Raman spectrum, g) X‐ray diffraction patterns, and h) FTIR spectra of P‐CDs (black line), Mn‐CDs (orange line), Zn‐CDs (purple line), Ni‐CDs (green line), and Cu‐CDs (blue line). i) High‐resolution Mn 2p spectra of Mn‐CDs. j) High‐resolution Zn 2p spectra of Zn‐CDs. k) High‐resolution Ni 2p spectra of Ni‐CDs. l) High‐resolution Cu 2p spectra of Cu‐CDs. Excitation‐emission mapping of (m) P‐CDs, n) Mn‐CDs, o) Zn‐CDs, p) Ni‐CDs, and q) Cu‐CDs. Optimal fluorescence excitation, and emission spectra of (r) P‐CDs, s) Mn‐CDs, t) Zn‐CDs, u) Ni‐CDs, and v) Cu‐CDs.

Next, the morphology and structure of these CDs were explored. The TEM images revealed that the sizes of P‐CDs, Mn‐CDs, Zn‐CDs, Ni‐CDs, and Cu‐CDs were 2.1, 2.8, 3.5, 4.2, and 4.4 nm, respectively (**Figures** **7**a–e; Figure S27, Supporting Information). These findings suggested that the incorporation of TMAs into the CDs matrix served as a bridge enlarging the sp^2^ domains of the CDs through TMAs─C/N/O bonds. Moreover, a higher doping quantity of TMAs resulted in a greater expansion of the sp^2^ domains. As shown in the Raman spectra (Figure 7f), the *I*_D_/*I*_G_ values for P‐CDs, Mn‐CDs, Zn‐CDs, Ni‐CDs, and Cu‐CDs were found to be 1.52, 1.34, 1.14, 0.91, and 0.87, respectively, indicating that the higher TMAs doping levels were associated with an increased graphite carbon content. The XRD patterns of all CDs showed a distinct peak located at 20–25°, consistent with the (002) graphite plane, and no peaks corresponding to the metals Mn, Zn, Ni, and Cu were observed (Figure 7g). The infrared spectra (Figure 7h) indicated that as the TMAs content increased, the absorption peaks at ≈3200 cm^−1^ associated with ─OH and ─NH₂ groups exhibited a decrease in intensity, signifying a reduction in these functional groups. Additionally, the signal intensity at 1388 cm^−1^, attributed to the bending vibration absorption of ─OH in O═C─O, was also decreased, suggesting a reduction in O═C─O content.^[^ ^23^ ^]^ The valence states of Mn, Zn, Ni, and Cu were further investigated through XPS. The Mn 2p spectrum (Figure 7i) displayed a satellite peak at 644.8 eV and a peak at 641.5 eV (Mn 2p_3/2_), indicating the presence of the +2 valence state of Mn, consistent with Mn─O bonds.^[^ ^19^ ^]^ Similarly, the Zn 2p spectrum (Figure 7j) exhibited a peak at 652.6 eV for Zn 2p_3/2_, confirming the +2 oxidation state of Zn related to Zn─O bonds.^[^ ^49^ ^]^ The Ni 2p spectrum (Figure 7k) showed a satellite peak at 861.1 eV and a peak at 855.5 eV for Ni 2p_3/2_, attributed to Ni─O bonds.^[^ ^50^ ^]^ For the Cu 2p spectrum (Figure 7l), peaks at 932.7 and 952.5 eV were assigned to Cu 2p_3/2_ and Cu 2p_1/2_, respectively, indicating the presence of Cu─O bonds in Cu‐CDs.^[^ ^51^ ^]^ Collectively, this evidence confirmed that TMAs─O bonds were successfully incorporated into CDs. The TMAs doping effectively decreased the oxidation degree of CDs.

Subsequently, the fluorescence properties of P‐CDs, Mn‐CDs, Zn‐CDs, Ni‐CDs, and Cu‐CDs were comprehensively investigated. It was found that the fluorescence emission intensity of TMAs‐CDs was significantly greater than that of P‐CDs under the same conditions. The excitation‐emission mapping revealed that the P‐CDs had excitation centers at 365 nm with maximum emission excitation centers ≈428 nm (Figures 7m,r). The Mn‐CDs displayed similar excitation and emission characteristics as P‐CDs, maintaining the same peaks at 365 and 428 nm, respectively (Figures 7n,s). The Zn‐CDs showed an emission center at ≈440 nm with an optimal excitation wavelength of 370 nm (Figures 7o,t). The Ni‐CDs demonstrated an emission center at 440 nm but had a slightly different excitation peak at 360 nm (Figures 7p,u). Moreover, the Cu‐CDs had optimal excitation and emission centers at 370 and 435 nm, respectively (Figures 7m,r). Additionally, we quantified the PLQY values of the above‐mentioned CDs. The P‐CDs exhibited a PLQY of 4.2%, while the Mn‐, Ni‐, Cu‐, and Zn‐CDs showed significantly higher values of 10.3%, 25.7%, 36.4%, and up to 51.6%, respectively (Table S5, Supporting Information). These results indicate that constructing TMAs─O bonds is one effective strategy for enhancing the PLQY of water‐soluble CDs.

## 3 Conclusions

In summary, we successfully synthesized Mn‐atomically‐doped red‐emitting carbon dots, described as R‐Mn‐CDs, featured with 41.3% PLQY in an aqueous environment, high stability, minimal cytotoxicity, and fluorescence/MR imaging bio‐imaging capability. Comprehensive investigations revealed that the doping of Mn atoms, configured in Mn‐N_2_O_2_ structure, resulted in the expanded sp^2^ domains, mitigated oxidation degree, narrowed bandgap of R‐Mn‐CDs, and suppressed non‐radiative relaxation channel for excited‐state electrons. Furthermore, the DFT calculations revealed the electron transfer from Mn atoms to N and O atoms and the effects of size, oxidation defects, and Mn‐N_2_O_2_ sites on the electronic structure of R‐Mn‐CDs. Moreover, successful in vivo fluorescence/MR dual‐mode imaging in mice was achieved. Finally, the construction of TMAs─O bonds was proven to be a universal strategy for enhancing the PLQY of water‐soluble CDs. Our findings not only clarify the mechanisms behind fluorescence enhancement through TMAs doping but also provide valuable theoretical insights for developing CDs with high PLQY.

## Conflict of Interest

The authors declare no conflict of interest.

## Supporting information



Supporting Information

## Data Availability

The data that support the findings of this study are available from the corresponding author upon reasonable request.
