# Supplementary material for: Elucidating Manganese Single‐Atom Doping: Strategies for Fluorescence Enhancement in Water‐Soluble Red‐Emitting Carbon Dots and Applications for FL/MR Dual Mode Imaging
Source: Adv Sci (Weinh). 2025 Jan 2;12(8):2414895. doi: 10.1002/advs.202414895 (PMC11848584; doi:10.1002/advs.202414895)
Supplement: Supplementary file 1 — Supporting Information [file ADVS-12-2414895-s001.docx]

**Supplementary Information**

Elucidating Manganese Single-Atom Doping: Strategies for Fluorescence Enhancement in Water-Soluble Red-Emitting Carbon Dots and Applications for FL/MR Dual Mode Imaging

**Author list:** Fucheng Gao^1, #^, Qiang Fu^2, 3 #^, Ying Ruan^4^, Can Li^1^, Yandong Wang^1^, Hui Li^1^, Jichao Li^5^, and Yanyan Jiang^1,^ *

**Affiliations:**

1. Key Laboratory for Liquid-Solid Structural Evolution and Processing of Materials, Ministry of Education, Shandong University, Jinan, 250061, China

2. Department of Urology, Shandong Provincial Hospital Affiliated to Shandong First Medical University, Jinan, China

3. Key Laboratory of Urinary diseases in Universities of Shandong (Shandong First Medical University)

4. MOE Key Laboratory of Materials Physics and Chemistry Under Extraordinary Conditions, School of Physical Science and Technology, Northwestern Polytechnical University, Xian, China

5. School of Physics Shandong university, Jinan, 250100, China

**Experimental Procedures**

**Materials and Reagents**

o-Phenylenediamine (OPD), Phytic acid (PA), Ethylenediaminetetraacetic acid (EDTA), Ethanolamine, MnCl_2_, AlCl_3_, ZnCl_2_, FeCl_3_, CuCl_2_, NiCl_2_, CaCl_2_, CoCl_2_, NaCl, and KCl were purchased from McLean. The 1000 Dalton cellulose dialysis bags were purchased from Shanghai yuanye Bio-Technology Co., Ltd. The ultrafiltration tubes with a molecular weight cutoff of 3 kDa were purchased from Millipore. The PC-3 cell lines, Ham’s F-12K, and Phosphate Buffered Saline (PBS) were purchased from Procell Life Science & Technology, Wuhan, China. The Llyso-Tracker Green, Mito-Tracker Green, Hematoxylin and Eosin Staining Kit (H&E), and Hochest were purchased from Beyotime Biotechnology, Shanghai, China. The chlorpromazine, filipin, amiloride, sodium azide, and phalloidin were purchased from Beijing Solarbio Science & Technology Co.,Ltd. The Fetal bovine serum (FBS) were purchased from XP Biomed, Shanghai, China, the distributors of Biological Industries, Israel. The Matrigel was purchased from Yeasen Biotechnology, Shanghai, China. The Cell Counting Kit-8 was purchased from Dojindo Molecular Technologies, Japan.

**Synthesis of R-Mn-CDs**

To prepare the R-Mn-CDs, 50 mg OPD and 1 mmol phytic acid (PA) were completely dissolved in 13 mL ultrapure water. Then, 2 mL of MnCl_2_ solution (0.5 mmol) was dropwise added to the above solution. After stirring adequately for 30 minutes, the resulting mixture was transferred to a 30 mL quartz bottle. The reaction was conducted in a microwave reactor (Monowave 200 purchased from Anton Paar) at a temperature of 180℃ for 4 hours. It should be noted that the microwave reactor employed in this study differs significantly from a conventional microwave oven, notably in its inability to modulate microwave power during the reaction. This apparatus relies on microwaves for heating, with material synthesis being regulated through precise control over temperature and duration. Consequently, the microwave power within the experimental setup undergoes dynamic variations during the reaction process. Upon reaction completion, the obtained solution underwent centrifugation to remove large impurities. For further purification, the solution of R-Mn-CDs was dialyzed in a 1000 Da dialysis bag for 24 hours, with water refreshed on an hourly basis. Ultimately, lyophilization resulted in approximately 53 mg of R-Mn-CDs powder, corresponding to a yield of 6.5%.

**Synthesis of Y-CDs**

The synthesis process for Y-CDs was identical to that of R-Mn-CDs, except that neither PA nor MnCl_2_ was added during the preparation of the reaction solution for Y-CDs. All other synthetic steps remain unchanged.

**Synthesis of R-CDs**

The synthesis process for R-CDs was also analogous to that of R-Mn-CDs. However, the distinct difference was that no MnCl_2_ solution was incorporated during the solution preparation of R-CDs. Again, all other steps in the synthesis process were the same as those used in the production of R-Mn-CDs.

**Synthesis of L-Arg@R-Mn-CDs**

To synthesize L-Arg@R-Mn-CDs, 10 mg of R-Mn-CDs and 50 mg of L-arginine (L-Arg) were dissolved in 5 mL of ultra-purified water. The mixture was then heated to 50 °C for one hour while being agitated. Following the reaction, the solution underwent dialysis using a 500 Da dialysis tube to eliminate any unreacted L-Arg molecules. Finally, the resulting solution was freeze-dried to yield the product, L-Arg@R-Mn-CDs.

**Optimization of Synthesis of Y-CDs, R-CDs, and R-Mn-CDs**

The optimization process for Y-CDs, R-CDs, and R-Mn-CDs involved reaction conditions and purification methods similar to those used in the preparation of the aforementioned CDs, with variations only in the ratios of the raw materials. Specifically, the CDs synthesized using 50 mg of OPD combined with PA at the amounts of 0.25 mmol, 0.5 mmol, 0.75 mmol, and 1 mmol as precursors were referred to as 0.25-R-CDs, 0.5-R-CDs, 0.75-R-CDs, and 1-R-CDs, respectively. Meanwhile, 0-R-Mn-CDs, 0.25-R-Mn-CDs, 0.5-R-Mn-CDs, and 1-R-Mn-CDs were synthesized by maintaining a constant amount of OPD at 50 mg and PA at 1 mmol while varying the addition of MnCl_2_ at the amounts of 0 mmol, 0.25 mmol, 0.5 mmol, and 1 mmol, respectively.

**Stability Test of Y-CDs, R-CDs, and R-Mn-CDs**

Y-CDs, R-CDs, and R-Mn-CDs solution (0.2 mg mL^-1^) were mixed with different metallic salt solutions (0.5 mM AlCl_3_, 0.5 mM ZnCl_2_, 0.5 mM FeCl_3_, 0.5 mM MnCl_2_, 0.5 mM NiCl_2_, 0.5 mM CaCl_2_, 0.5 mM, CoCl_2_, 0.5 mM NaCl and 0.5 mM KCl). Following a 5-minute incubation period at 37 °C, fluorescence measurements were conducted. For Y-CDs, the fluorescence intensity was recorded at 570 nm using a laser excitation wavelength of 420 nm. The fluorescence of both R-CDs and R-Mn-CDs was measured at 625 nm under excitation with a laser set to 610 nm. To assess the photostability of the samples, a 420 nm laser was employed for illuminating Y-CDs and a 610 nm laser for R-CDs and R-Mn-CDs. The continuous illumination lasted for 60 minutes. During this period, the fluorescence intensity of Y-CDs (0.2 mg mL^-1^) was monitored at an emission wavelength of 570 nm. Simultaneously, the fluorescence intensity of both R-CDs and R-Mn-CDs at a concentration of 0.2 mg mL^-1^ was recorded at an emission wavelength of 625 nm. Subsequently, the fluorescence intensity of Y-CDs, R-CDs, and R-Mn-CDs (0.2 mg mL^-1^) was measured at different temperatures (10-90 °C) and pH values (2-12). All stability tests were set up in three sets of parallel experiments. These fluorescence measurements were conducted using a Shimadzu RF-6000 spectrophotometer in Japan.

**DFT Calculations**

Initially, the Vienna *Ab initio* Simulation Package (VASP) was employed to compute the formation energy (E_f_) of the three configurations (I-Mn-N_2_O_2_, II-Mn-N_2_O_2_ and III-Mn-N_2_O_2_). Specifically, the spin-polarized projected augmented wave (PAW) method is adopted^[1]^, and the Perdew-Burke-Ernzerhof (PBE) type of the generalized gradient approximation (GGA)^[2,3]^ is applied as exchange-correlation energy functional. For all calculations, the plane-wave cutoff energy is set to 500 eV, and the Grimme’s D3 correction is employed to account for dispersion interaction. The total energy is converged to less than 10^-4^ eV, while the atomic force is converged to less than 0.02 eV/Å. The Brillouin zone is sampled by a Gamma centered 2 × 2 × 1 K-points mesh. The configurations model is constructed by doping the Mn-N_2_O_2_ moiety into the surface layer of a three-layer graphene supercell with surface periodicity of 6 × 6 A vacuum slab of 15 Å was generated along the z axis to ensure negligible interaction between mirror images.

The *E*_f_ is defined as^[4]^,

| $E_{f}=E_{\text{tot}}-\sum_{i} n_{i}\mu_{i}$ | (3) |
| --- | --- |

where *E*_tot_ is the total energy of the configuration, *n_i_* is the quantity of type *i* atoms in the catalyst, and *μ_i_* is the chemical potential of type *i* atom.

The differential charge density is calculated as follows,

| $\Delta\rho=\rho_{\mathrm{configuration}}-\rho_{configuration-\text{Mn}}-\rho_{\text{Mn}}$ | (4) |
| --- | --- |

where *ρ**_configuration_*, *ρ_configuration_*_-Mn_ and *ρ*_Mn_ denote the charge densities of the relaxed configuration, configuration without the Mn atom and single Mn atom, respectively.

Furthermore, the B3LYP density functional method with D3(BJ)^[5]^ dispersion correction was employed in this work to calculate the optimal conformations and bandgaps of the possible four molecules (I-Y-CDs, II-R-CDs, III-R-CDs, and IV-R-Mn-CDs). For geometry optimization and frequency calculations, SDD basis set^[6,7]^ was used for Mn and 6-31G (d, p) for others^[8,9]^. The Gaussian 16 suite of programs was used throughout this study.

**Preparation of Different TMAs-doped CDs (TMAs=Mn, Zn, Ni, and Cu)**

For example, the Cu-CDs were synthesized by hydrothermal method utilizing EDTA, ethanolamine, and CuCl_2_ as precursors. Specifically, 2 mmol of EDTA and 2 mmol of ethanolamine were combined with 0.5 mmol of CuCl_2_ in 20 mL of ultrapure water. This mixture was stirred at room temperature for 30 minutes to ensure thorough mixing. Subsequently, the solution was transferred into a Teflon-lined autoclave with a capacity of 50 mL and heated to 180 ℃ for a duration of 10 hours. After the reaction period, the system was allowed to cool down to room temperature. To isolate the Cu-CDs, the solution was filtered through a polyether sulfone membrane with a pore size of 0.22 µm to eliminate larger particles. The resulting solution underwent dialysis for 24 hours using ultrapure water in a dialysis bag with a molecular weight cutoff of 1000 Da. Finally, the purified Cu-CDs were lyophilized for further analysis. The preparation processes for Mn-CDs, Zn-CDs, and Ni-CDs followed the same protocol with different metal sources utilized. In the reaction system for Mn-CDs, 0.5 mmol of MnCl_2_ was incorporated. Similarly, for Zn-CDs, 0.5 mmol of ZnCl_2_ was added to the reaction mixture. In the case of Ni-CDs, 0.5 mmol of NiCl_2_ was included in the reaction setup. During the preparation of P-CDs, only EDTA and ethanolamine were added, without the inclusion of any metal sources.

**Cell Culture**

The human cell lines PC-3 was obtained from Procell Life Science & Technology (Wuhan, China) and cultured with Ham’s F-12K (Kaighn’s) (F-12K) (Gibco, MA, USA) medium with 10% fetal bovine serum (FBS) (Biological Industries, Israel) in a CO_2_ incubator at 37 °C.

**Cell Proliferation Assays**

The PC-3 cells were first seeded in the 96-well plate (1×10^4^ cells per well) and cultured for 24 h. Then, F-12K containing R-Mn-CDs at various concentrations (0, 50, 100, and 200 μg mL^-1^) was added to each well to replace the cell culture media and cultured for 24 hours. After that, the plates were washed three times using PBS solution. Subsequently, the cells were incubated with 100 μL of F-12K contained 10 μL CCK-8 solution for 1 h. After incubation process, the optical density was recorded by microplate reader at the wavelength of 450 nm.

Annexin V-FITC apoptosis detection kit is used to measure the apoptosis of PC-3 cells with different concentrations of R-Mn-CDs (0, 50, 100, and 200 µg mL^-1^) for 72 hours. Subsequently, PC-3 cells were stained with Annexin V-FITC/PI Apoptosis Detection Kit according to the manufacture instruction and analyzed using flow cytometry. At least 10000 events were acquired for each group.

**Animals’ Xenograft Experiment**

All animal studies were approved by the Institutional Animal Care and Use Committee of Shandong Provincial Hospital (No.2023-036). 4-6 weeks old male BALB/c nude mice (Vital River Laboratory Animal Technology Co, Beijing, China) were used for animal experiments.

*In vivo* imaging was carried out using an IVIS 200 small-animal imaging system. Initially, a dosage of 10 mg kg^-1^ of R-Mn-CDs was administered to the mice via intraperitoneal injection. The imaging of the mice was conducted 5 minutes post-injection to evaluate the distribution and fluorescence characteristics of R-Mn-CDs. Subsequently, another group of mice received the same dosage of R-Mn-CDs through tail vein injection. To investigate the metabolic process of R-Mn-CDs, major organs including the heart, liver, spleen, lungs, and kidneys were harvested at various time points (0, 0.5, 1, 1.5, 2, 3, and 6 hours) for imaging analysis. Furthermore, these organs were collected and preserved in 4% paraformaldehyde for 24 h. Five-micrometer-thick sections were stained with hematoxylin and eosin (H&E).

The human prostate cancer cell line PC-3 was cultured until it reached the logarithmic growth phase. The cells were then digested and centrifuged under sterile conditions to create a cell suspension in PBS, with the cell count adjusted accordingly. A cell injection solution was prepared by mixing PBS and Matrigel (Yeasen Biotechnology, Shanghai, China) in a 1:1 ratio. The cells were injected subcutaneously into the front armpit of nude mice, with each mouse receiving 5×10^6^ cells in a volume of 100 μL. The cell suspension was injected using a 1 mL sterile syringe and needle. Following injection, the mice were returned to a sterile vivarium for observation of their recovery. The mice received continuous monitoring and care, with regular assessments of tumor growth. Once tumors were established, R-Mn-CDs (10 mg Kg^-1^) were injected either via tail vein or intratumoral injection. Magnetic Resonance Imaging (MRI) was then performed using a Small Animal MRI Imager.

To investigate renal metabolism of R-Mn-CDs, mice were intravenously administered with R-Mn-CDs, followed by collecting urine samples within the next 2-6 hours. The urine samples were subjected to ultrafiltration using 3 kDa cutoff tubes to remove low molecular weight proteins. Subsequently, the samples underwent a 6-hour dialysis against fresh water, with water exchanged every 30 minutes, utilizing a 1 kDa bag for further purification of small molecular substances. After dialysis, the urine was concentrated via rotary evaporation before being applied to an ultra-thin carbon film for transmission electron microscopy (TEM) analysis.

**Fluorescence Microscopy**

PC-3 cells were cultured at a density of 1×10^4^ per well in dishes and incubated at 37 °C with 5% CO₂ for 24 hours to achieve complete adherence. Subsequently, cells were treated with varying concentrations of R-Mn-CDs ranging from 0 to 200 µg mL^-1^ for 4 hours. Post incubation, the medium was discarded, and cells were thrice washed with PBS (pH 7.4) to eliminate any residual nanocomposite materials. The fixation process was initiated using a solution of 4% paraformaldehyde for 30 min. After fixation, the cells were washed three times with PBS again. A solution of 0.03% Triton X-100 was applied, followed by another series of three washes with PBS. Next, the cytoskeleton was stained using Actin-Tracker Green-488 (Beyotime C2201S) for 30 minutes before performing three additional washes with PBS. Finally, to preserve fluorescence during imaging, an anti-fade mounting medium containing DAPI (ABCAM AB104139) was used to cover the samples. Imaging was conducted using a laser scanning confocal microscope (Carl Zeiss CLSM 980).

For time-dependent imaging assays, PC-3 cells were seeded and cultured under the same conditions as previously described and then exposed to F-12K medium containing R-Mn-CDs at a concentration of 50 µg mL^-^¹ at intervals of 2, 6, 12, 24, and 48 hours. After each treatment duration, excess materials were removed through triple washes with PBS before proceeding through fixation and staining protocols as outlined earlier. The same imaging techniques were used to visualize these samples using CLSM.

**Study on the Mechanism of PC-3 Cells Absorbing R-Mn-CDs**

PC-3 cells were seeded in culture dishes with a cell density of 1×10^4^ per well, and cultured in a cell incubator with 5% carbon dioxide at 37 ℃ for 24 h. Next, PC-3 cells were incubated with different pathway inhibitors, including chlorpromazine (10 μg mL^-1^), filipin (10 μg mL^-1^), amiloride (50 μM) and sodium azide (10 mM) solution for 2 h. In addition, another group of PC-3 cells was treated at a low temperature of 4 °C for a duration of 2 hours. Then, R-Mn-CDs were added into the culture medium with final concentration of 100 μg mL^-1^ and cultured with PC-3 cells for 2 h. Afterwards, the cells were washed with PBS for three times. The fixation process was initiated using a solution of 4% paraformaldehyde for 30 mins. Post-fixation, the cells were washed three times with PBS again. To permeabilize the cell membranes, a solution of 0.03% Triton X-100 was applied, followed by another series of three washes with PBS. Next, the cytoskeleton was stained using Actin-Tracker Green-488 (Beyotime C2201S) for 30 minutes before performing three additional washes with PBS. Finally, to preserve fluorescence during imaging, an anti-fade mounting medium containing DAPI (ABCAM AB104139) was used to cover the samples. Imaging was conducted using a laser scanning confocal microscope (Carl Zeiss CLSM 980).

**Characterizations**

The morphology of Y-CDs, R-CDs, R-Mn-CDs, P-CDs, Mn-CDs, Zn-CDs, Ni-CDs, and Cu-CDs were obtained on transmission electron microscope (TEM; HT-7700, HITACHI and FEI Titan3 Cubed 60-300) and atomic force microscope (AFM; Bruker Dimension ICON). The X-ray diffraction spectra of these CDs were obtained using a Rigaku SmartLab SE. The Raman spectra were analyzed using the same Rigaku SmartLab SE instrument. Fourier transform infrared spectrometer (Nicolet 6700, Thermo Scientifuc) was used to analyze the functional group information of these samples. X-ray photoelectron spectroscopy (ESCALAB 250Xi, Thermo Electron, USA) was used to confirm the chemical state of these CDs. The pH meter used in the experiments is PHS-3C, INESA Scientific Instrument Co., Ltd., China. The ultraviolet-visible spectrum was measured by UV-Visible spectrophotometer (SPECORD 200 PLUS, Jena, Germany). The Zeta Potential was measured by the Malvern Zetasizer Nano ZS (Malvern Instruments Ltd., Worcestershire, UK). The EPR measurements were carried out via the Bruker EPR EMX Plus (Bruker Ltd, USA). The fluorescence lifetime and quantum yield of these CDs were ascertained with the Edinburgh Instruments FLS100 steady-state transient fluorescence spectrophotometer. The nuclear magnetic resonance hydrogen spectrum was determined by dissolving R-Mn-CDs in deuterium oxide, conducted using a Bruker 400 MHz spectrometer. The femtosecond transient absorption spectra of these CDs were measured on HELIOS, which was purchased from Ultrafast Systems. The scanning confocal fluorescence imaging microscope (CLSM800, Carl Zeiss, Germany) was applied to observe the cell uptake; cytotoxicity of these materials was determined by enzyme-labeled instrument (Infinite M200 Pro, Switzerland).

| 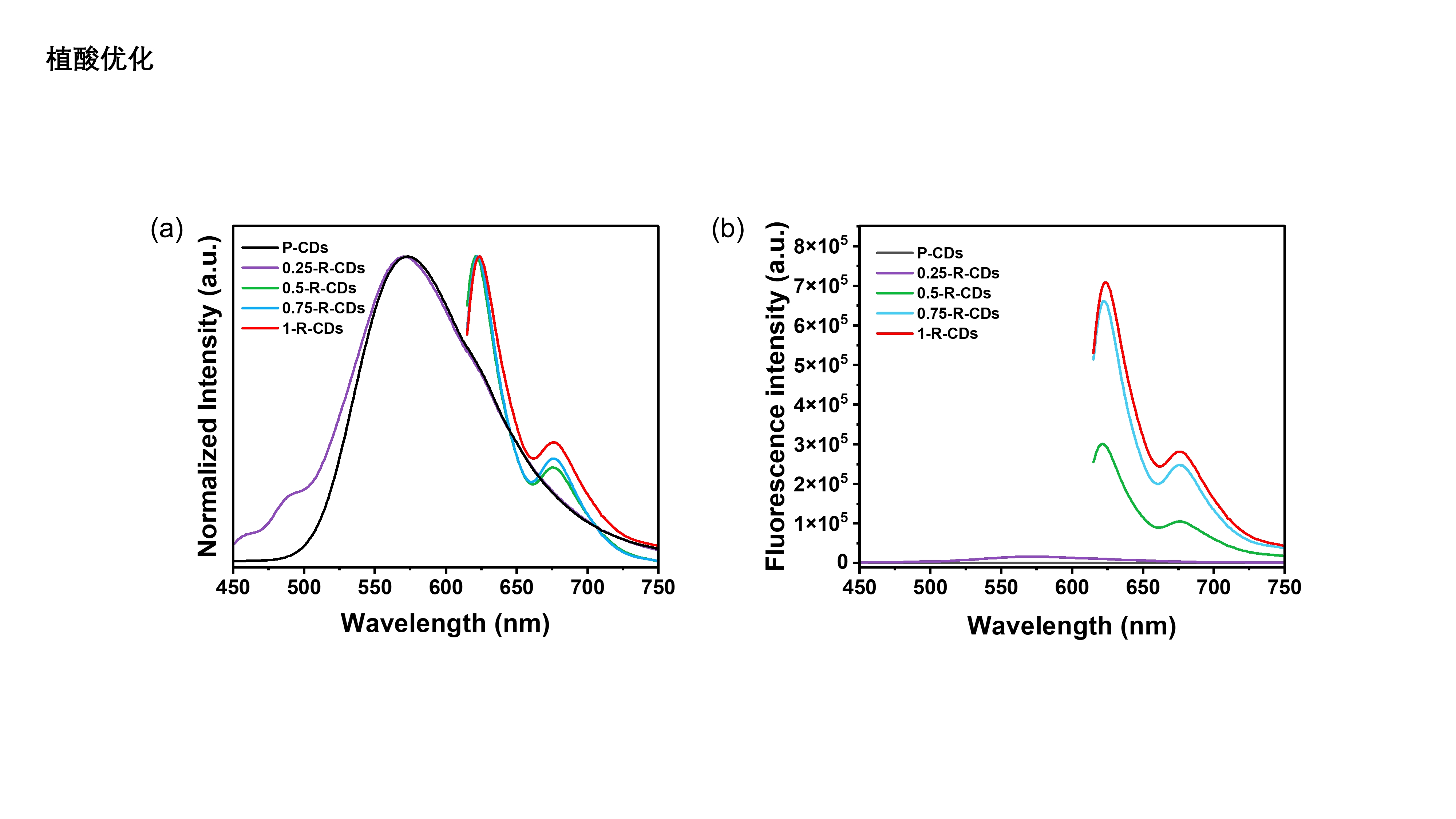 |
| --- |
| **Figure S1.** (a) Normalized fluorescence emission spectra of CDs prepared with different phytic acid concentrations. (b) Comparison of the fluorescence emission intensity of CDs prepared with different phytic acid concentrations. P-CDs refer to CDs that were prepared solely from OPD, while 0.25-R-CDs, 0.5-R-CDs, 0.75-R-CDs, and 1-R-CDs represented the R-CDs produced using PA in quantities of 0.25 mmol, 0.5 mmol, 0.75 mmol, and 1 mmol, respectively. |

| 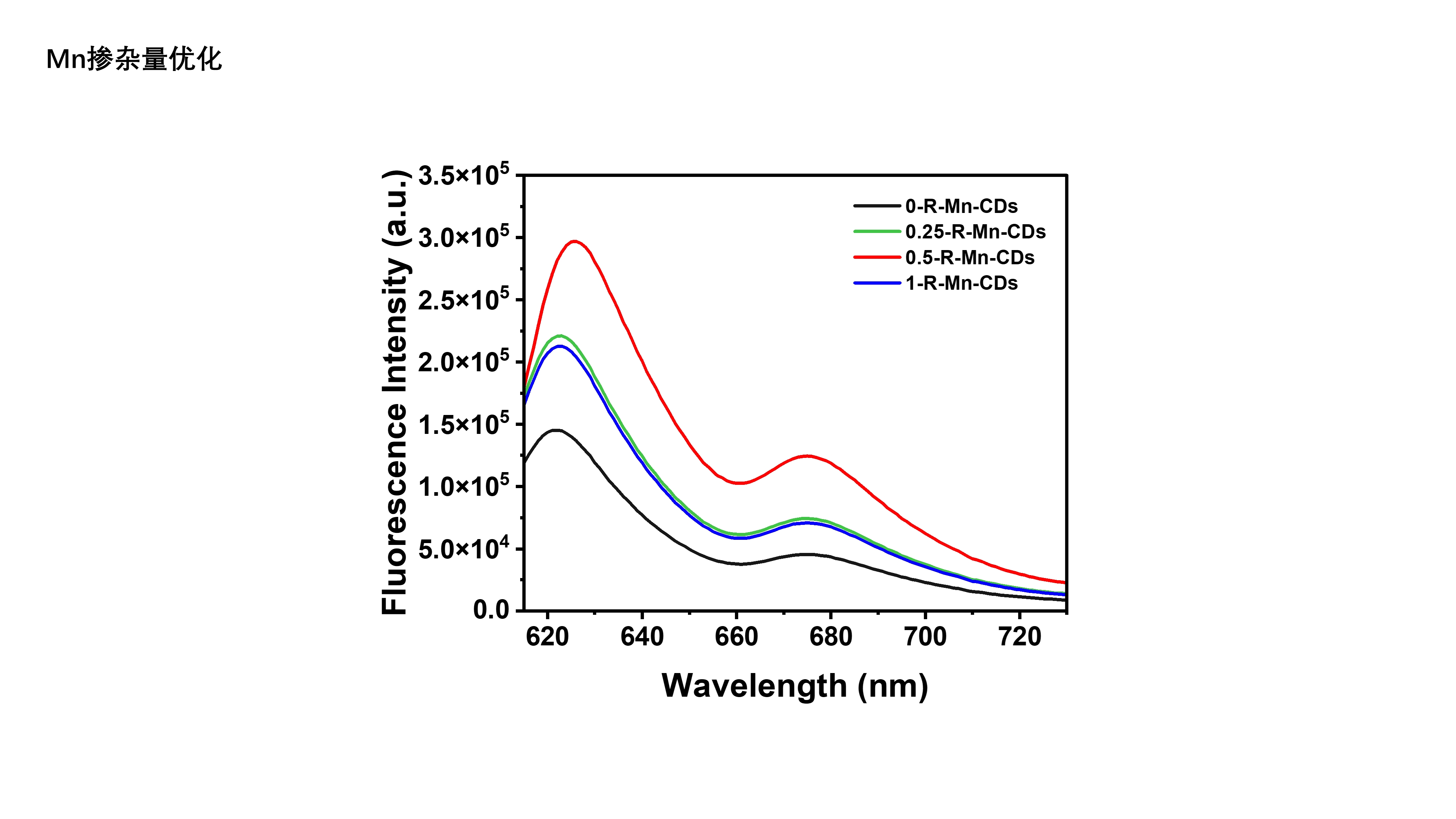 |
| --- |
| **Figure S2.** Comparison of the fluorescence emission intensity of R-Mn-CDs prepared with different addition of Mn ion. The designations of 0-R-Mn-CDs, 0.25-R-Mn-CDs, 0.5-R-Mn-CDs, and 1-R-Mn-CDs correspond to the R-Mn-CDs synthesized using Mn ion dosages of 0 mmol, 0.25 mmol, 0.5 mmol, and 1 mmol during the synthesis procedure. |

| 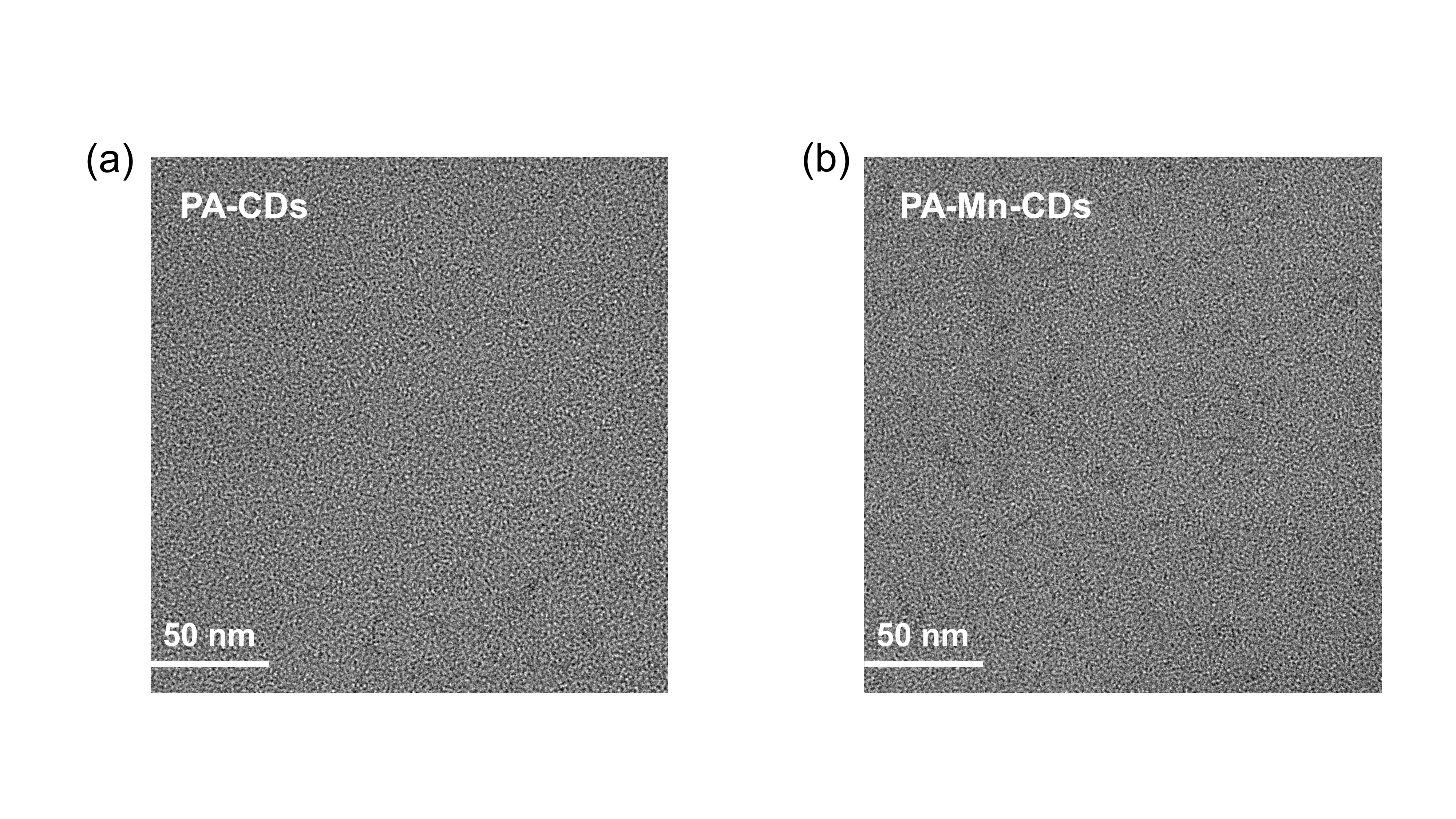 |
| --- |
| **Figure S3.** (a) TEM image of CDs prepared only with phytic acid as a carbon source. (b) TEM image of CDs prepared with a combination of phytic acid and Mn ions as a carbon source. There was the morphological structure of CDs observed in the TEM images, indicating that no CDs was obtained. |

| 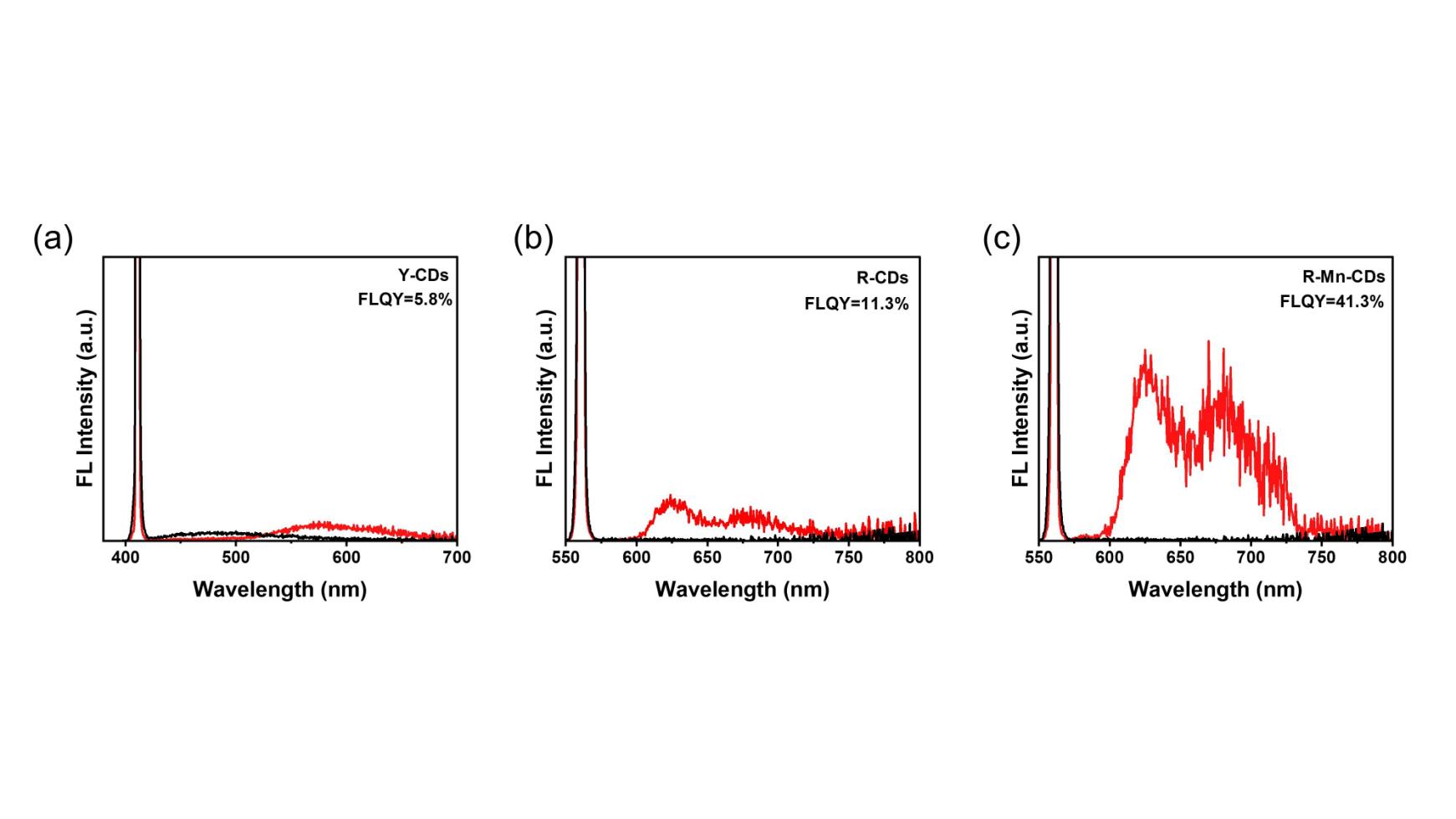 |
| --- |
| **Figure S4.** The absolute fluorescence quantum yield of (a) Y-CDs, and (b) R-CDs, and (c) R-Mn-CDs. |

| 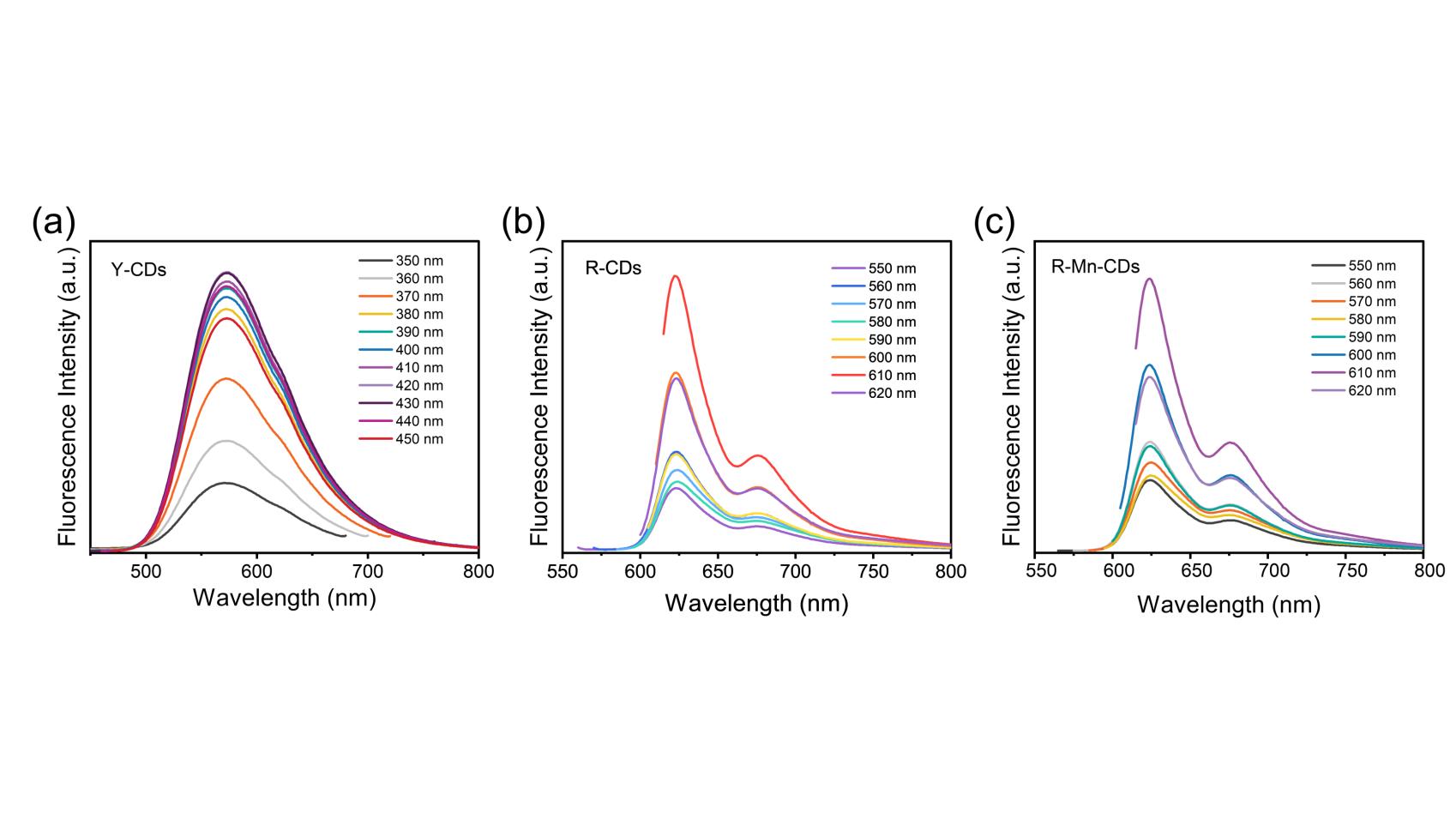 |
| --- |
| **Figure S5.** The fluorescence emission spectra of (a) Y-CDs, (b) R-CDs, and (c) R-Mn-CDs at different excitation wavelengths. |

| 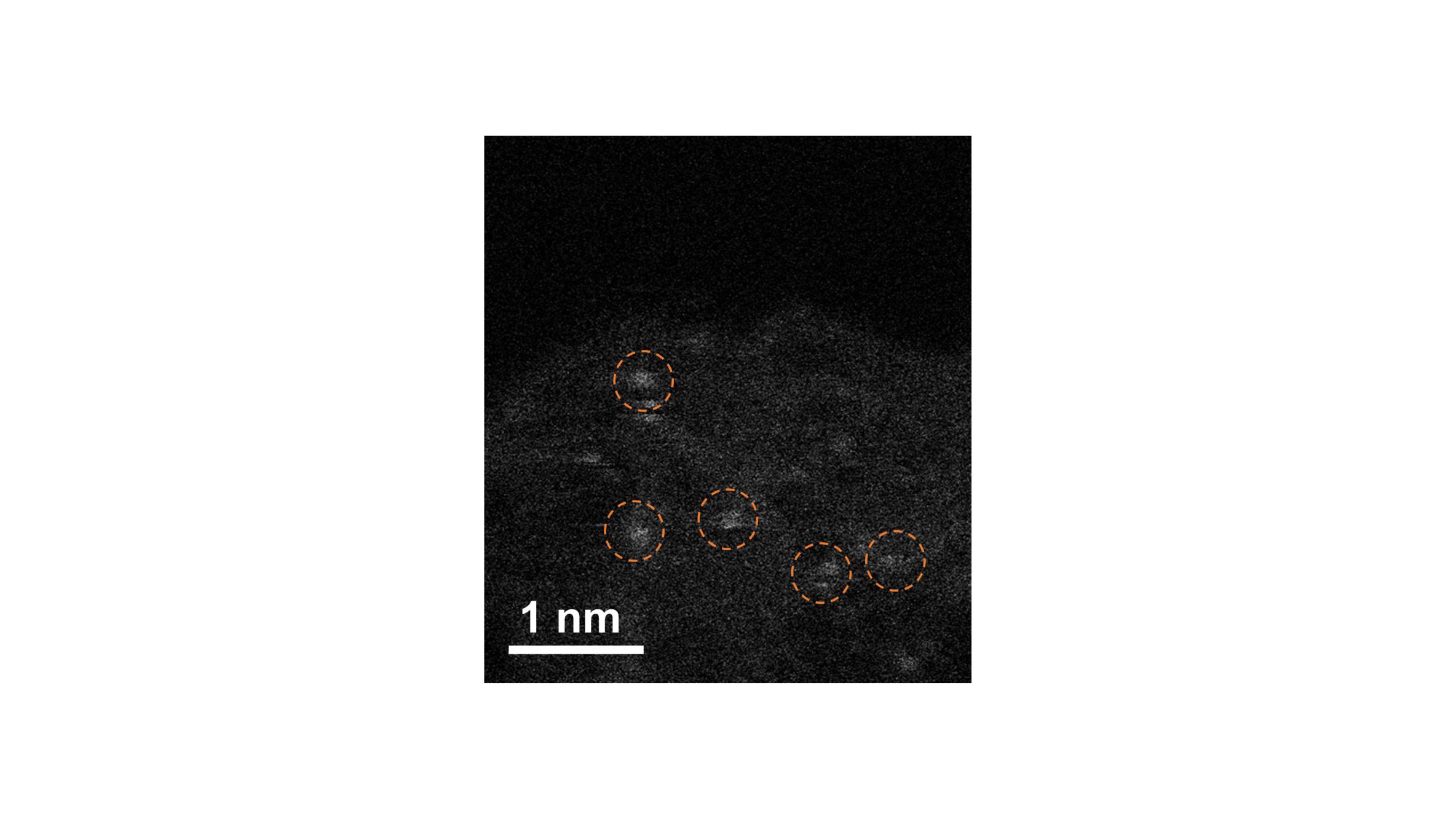 |
| --- |
| **Figure S6.** The STEM image of R-Mn-CDs. The yellow circles highlight single Mn atoms. |

| 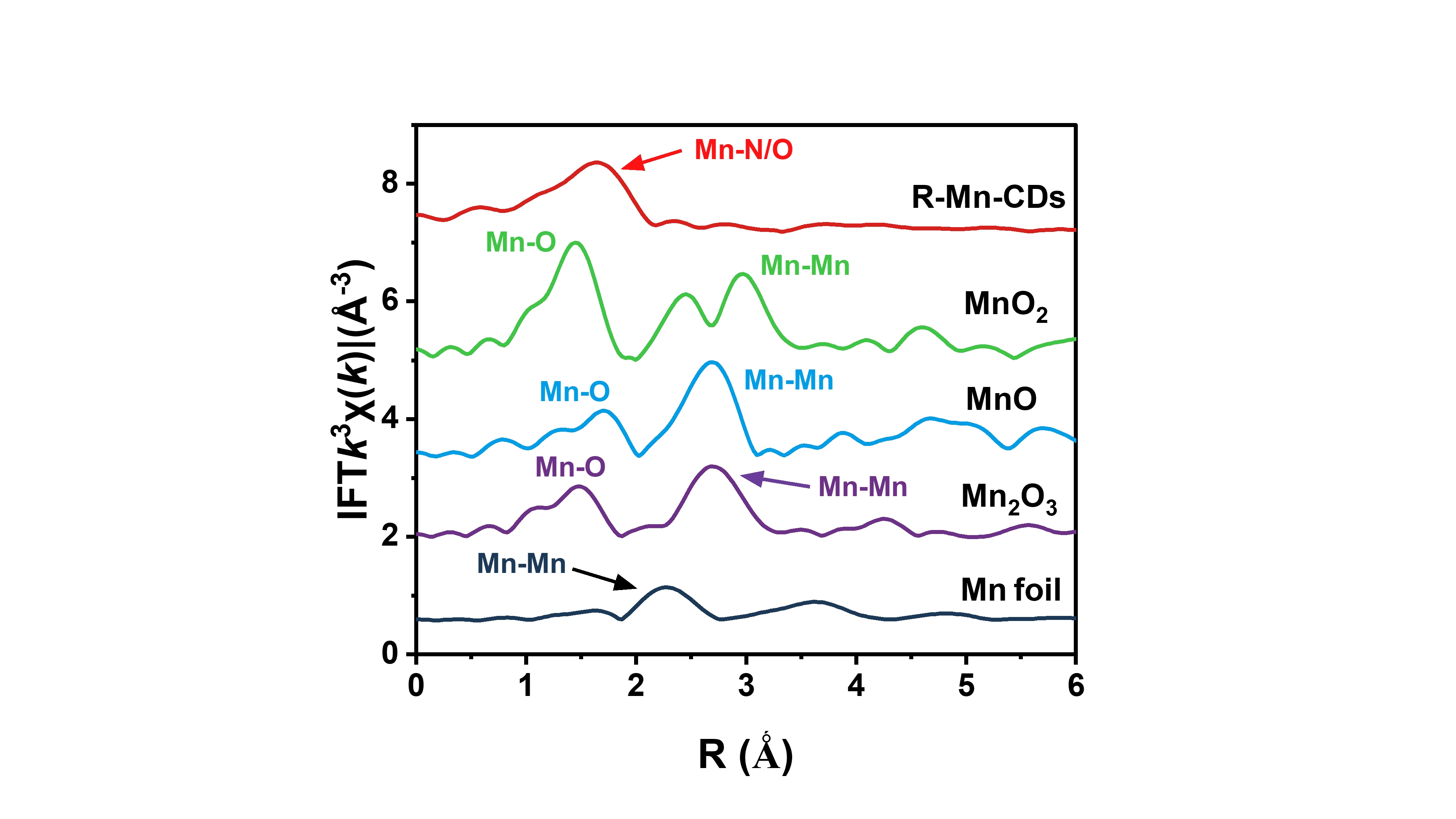 |
| --- |
| **Figure S7.** The FT-EXAFS at the Mn K-edge of R-Mn-CDs and reference samples. |

| 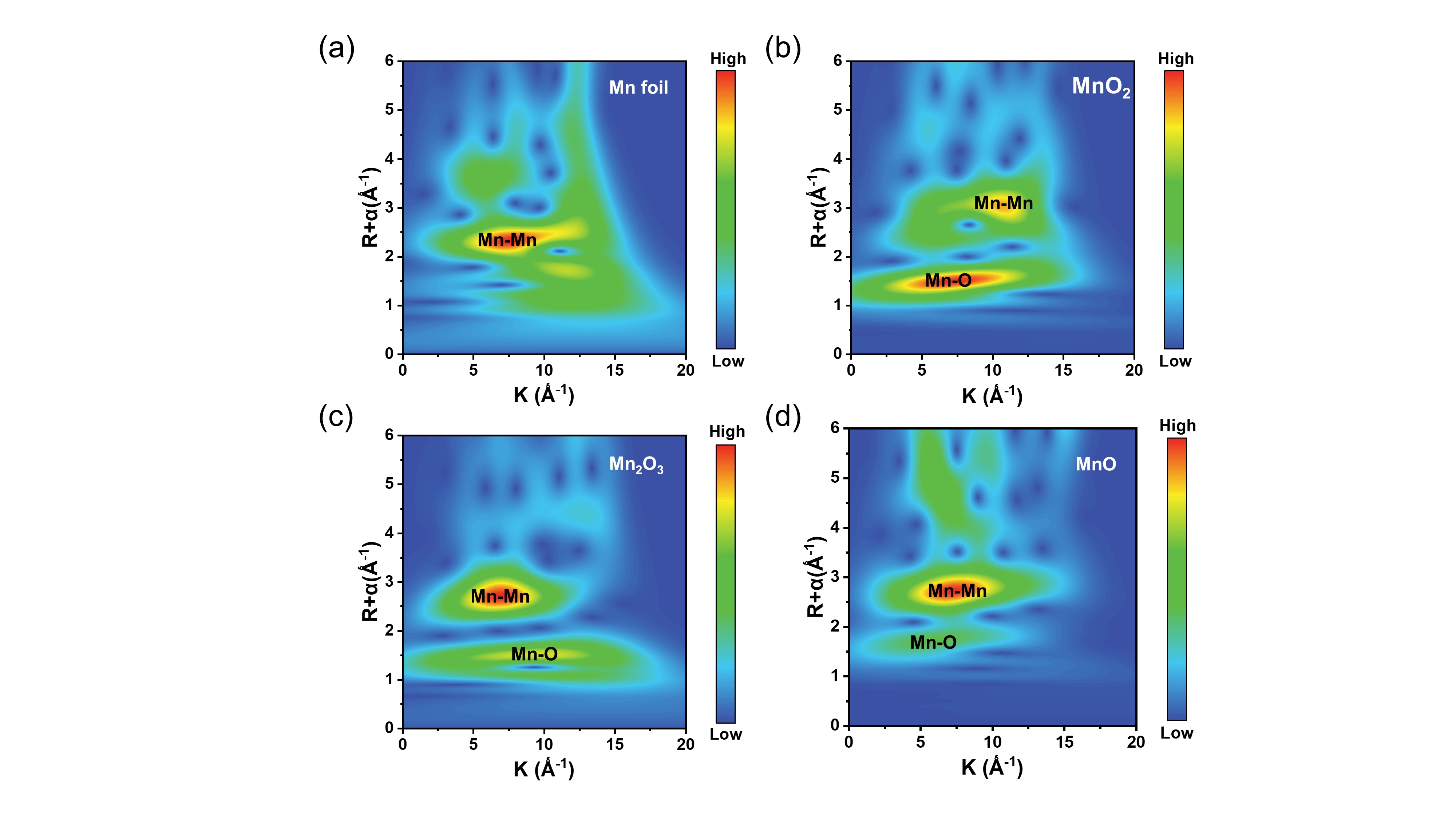 |
| --- |
| **Figure S8.** The wavelet transforms images at the Mn K-edge of (a) Mn foil, (b) MnO_2_, (c) Mn_2_O_3_, and (d) MnO. |

| 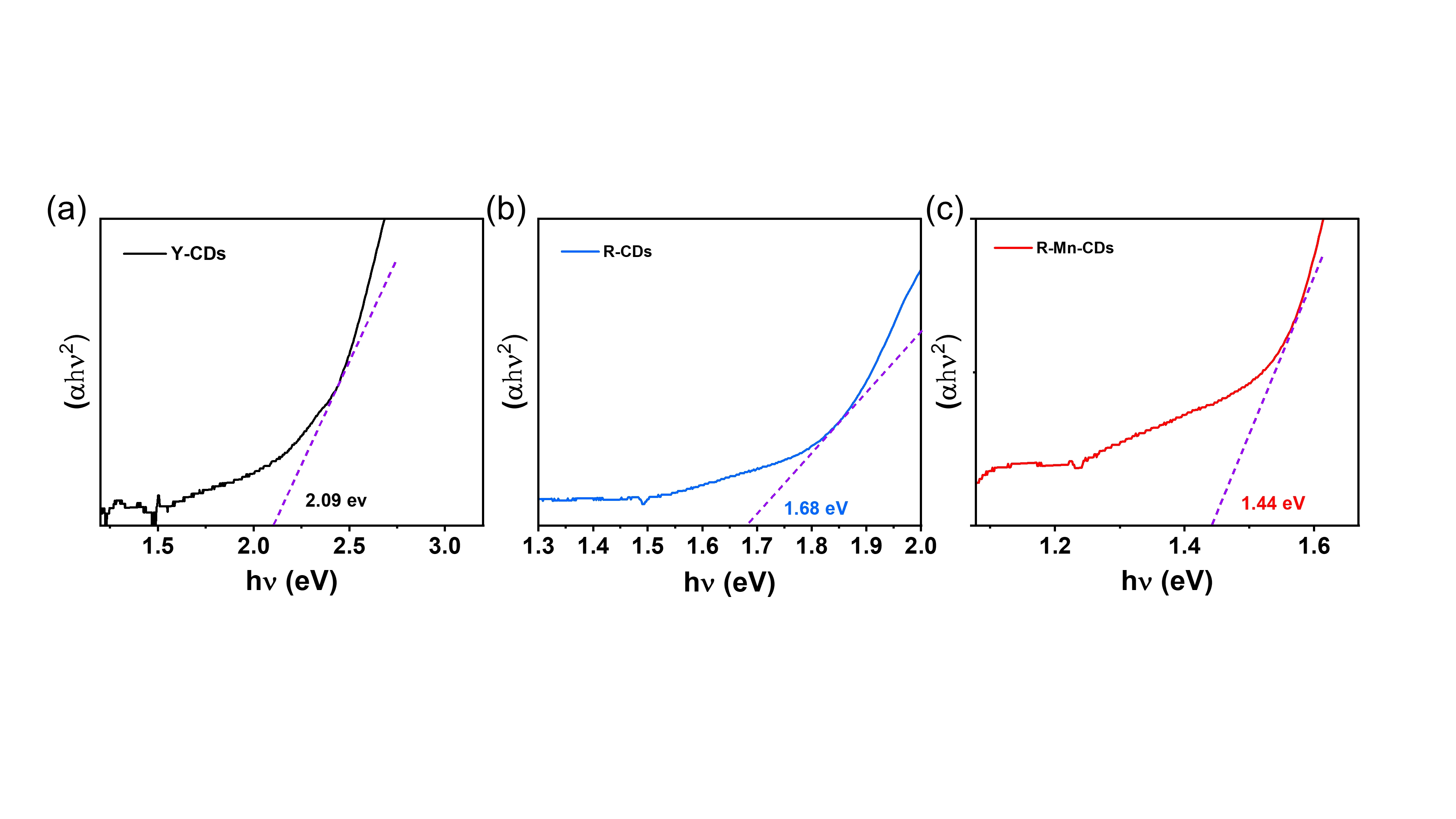 |
| --- |
| **Figure S9.** The tauc plot spectra of (a) Y-CDs, (b) R-CDs, and (c) R-Mn-CDs |

| 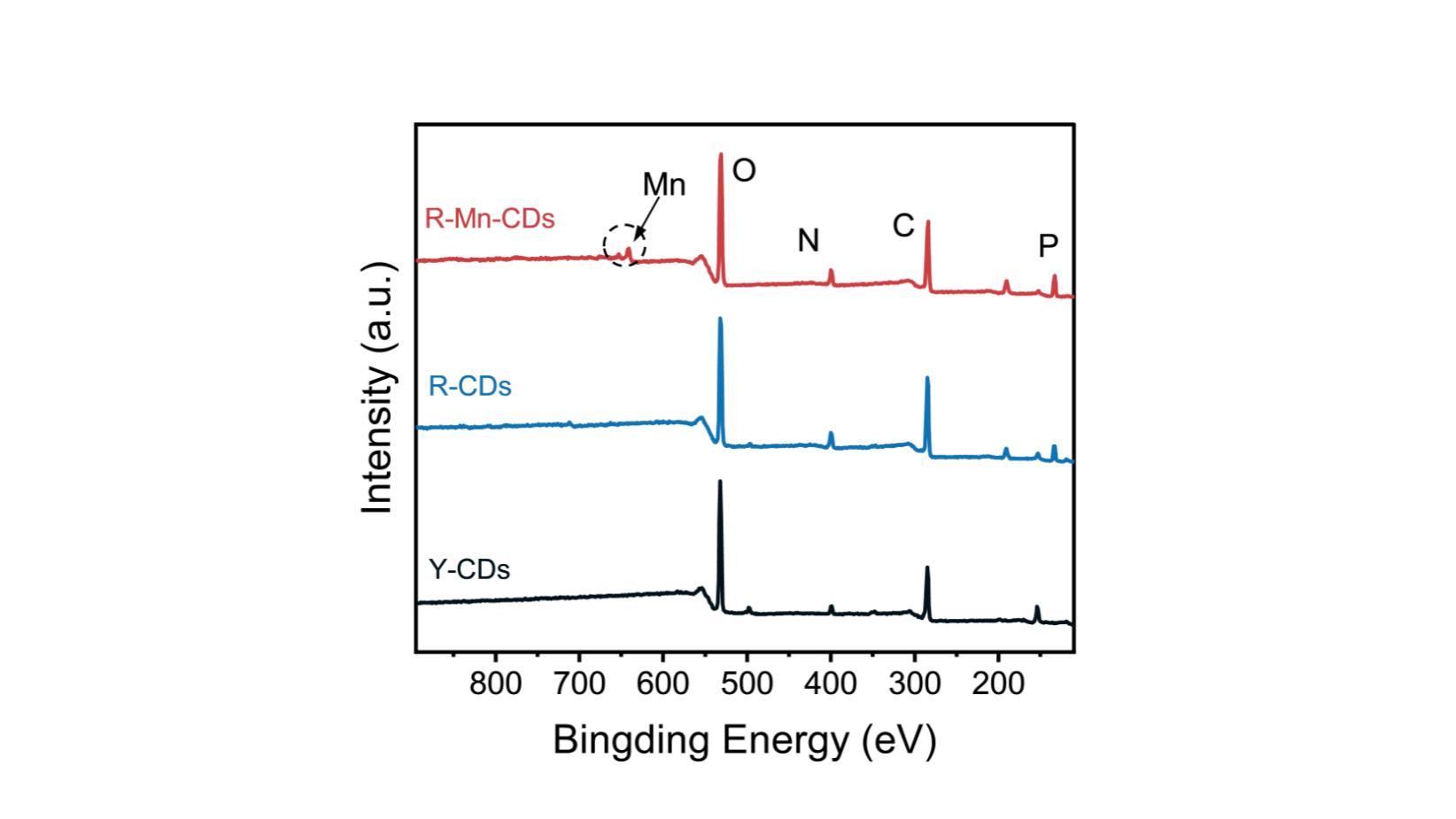 |
| --- |
| **Figure S10.** The survey scan XPS spectrum of Y-CDs (black line), R-CDs (bule line), and R-Mn-CDs (red line). |

| 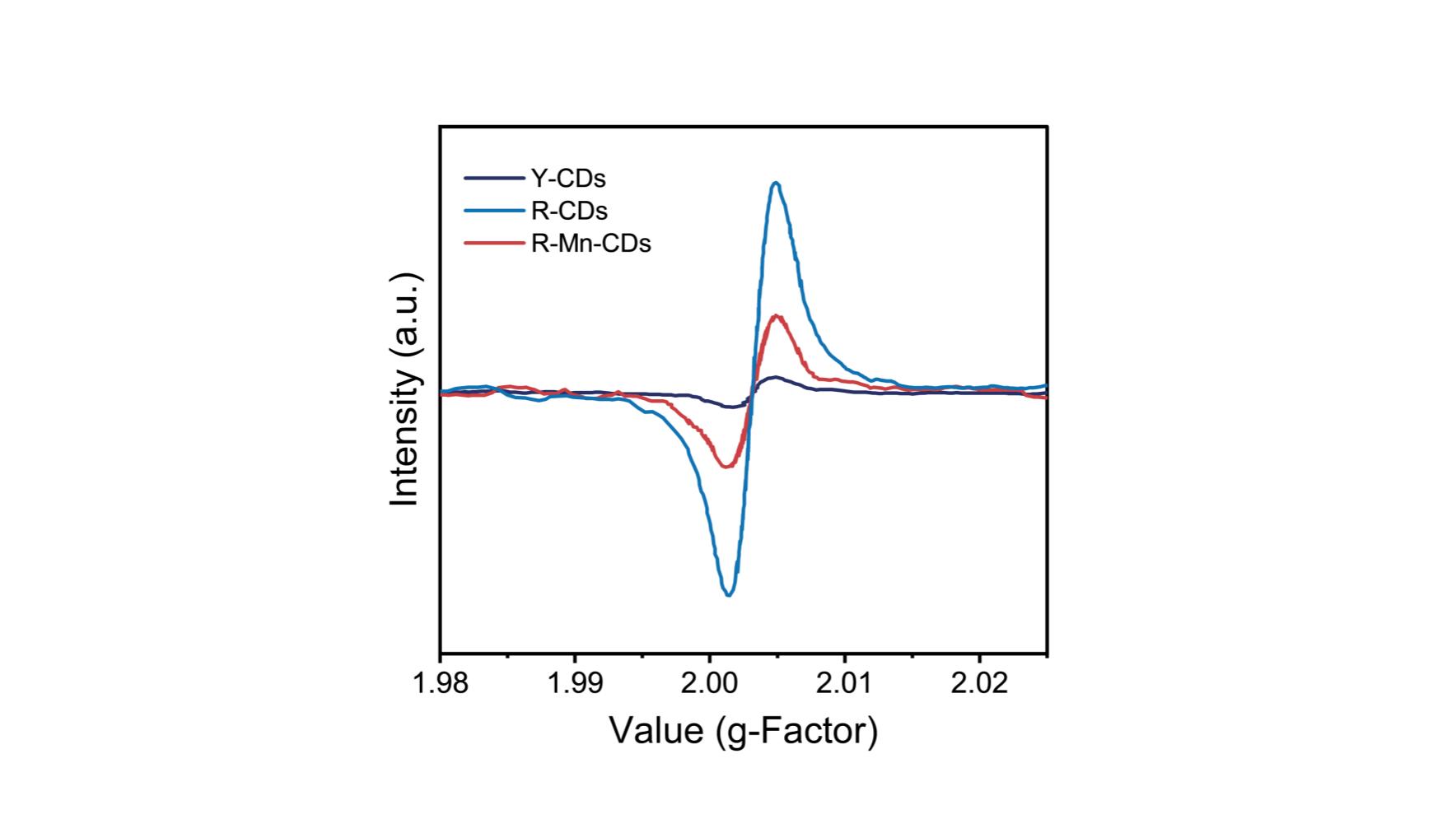 |
| --- |
| **Figure S11.** The EPR spectra of Y-CDs (black line), R-CDs (blue line), and R-Mn-CDs (red line). |

| 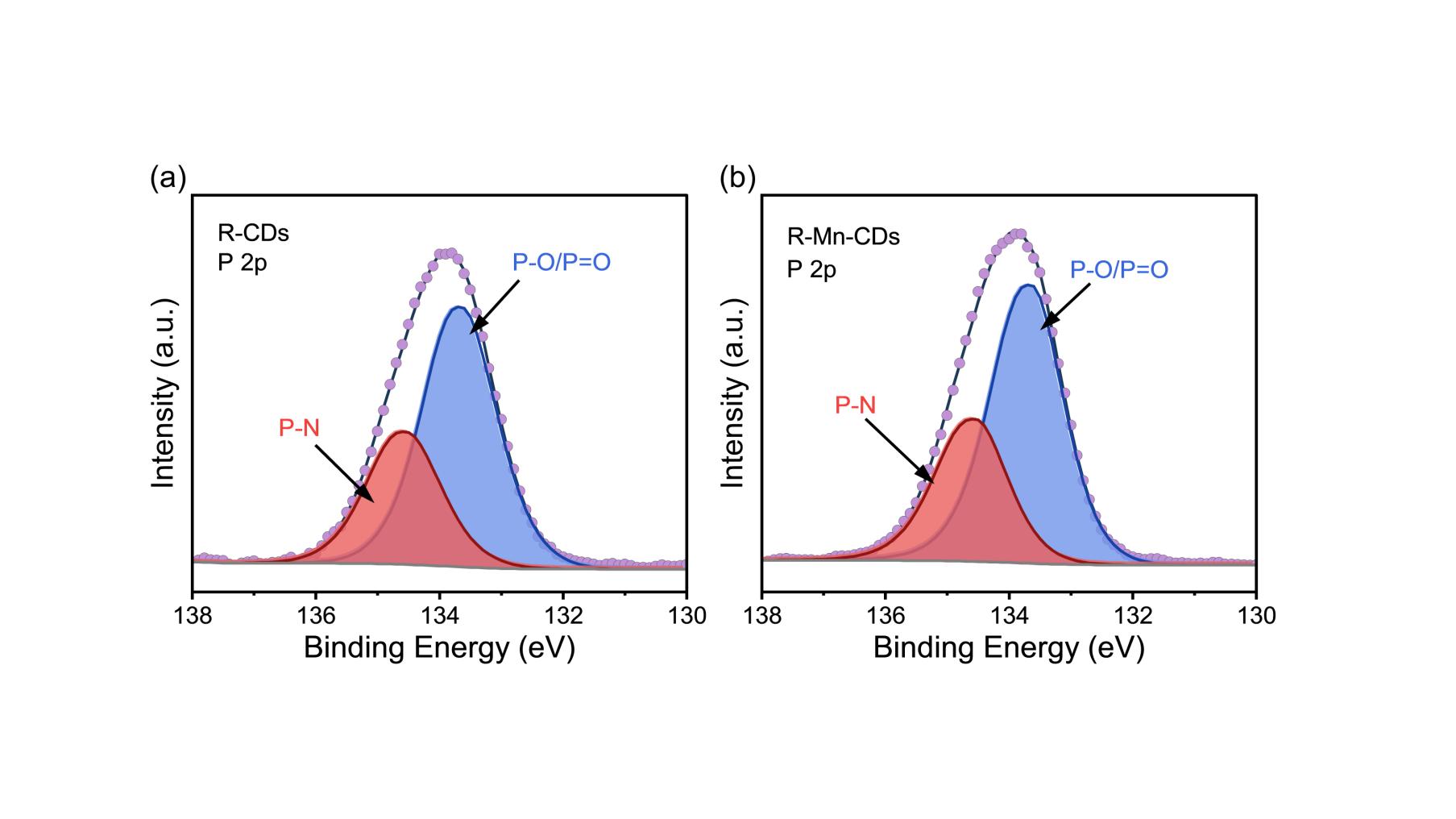 |
| --- |
| **Figure S12.** The high-resolution P 2p spectra of (a) R-CDs, and (b) R-Mn-CDs. |
|  |

| 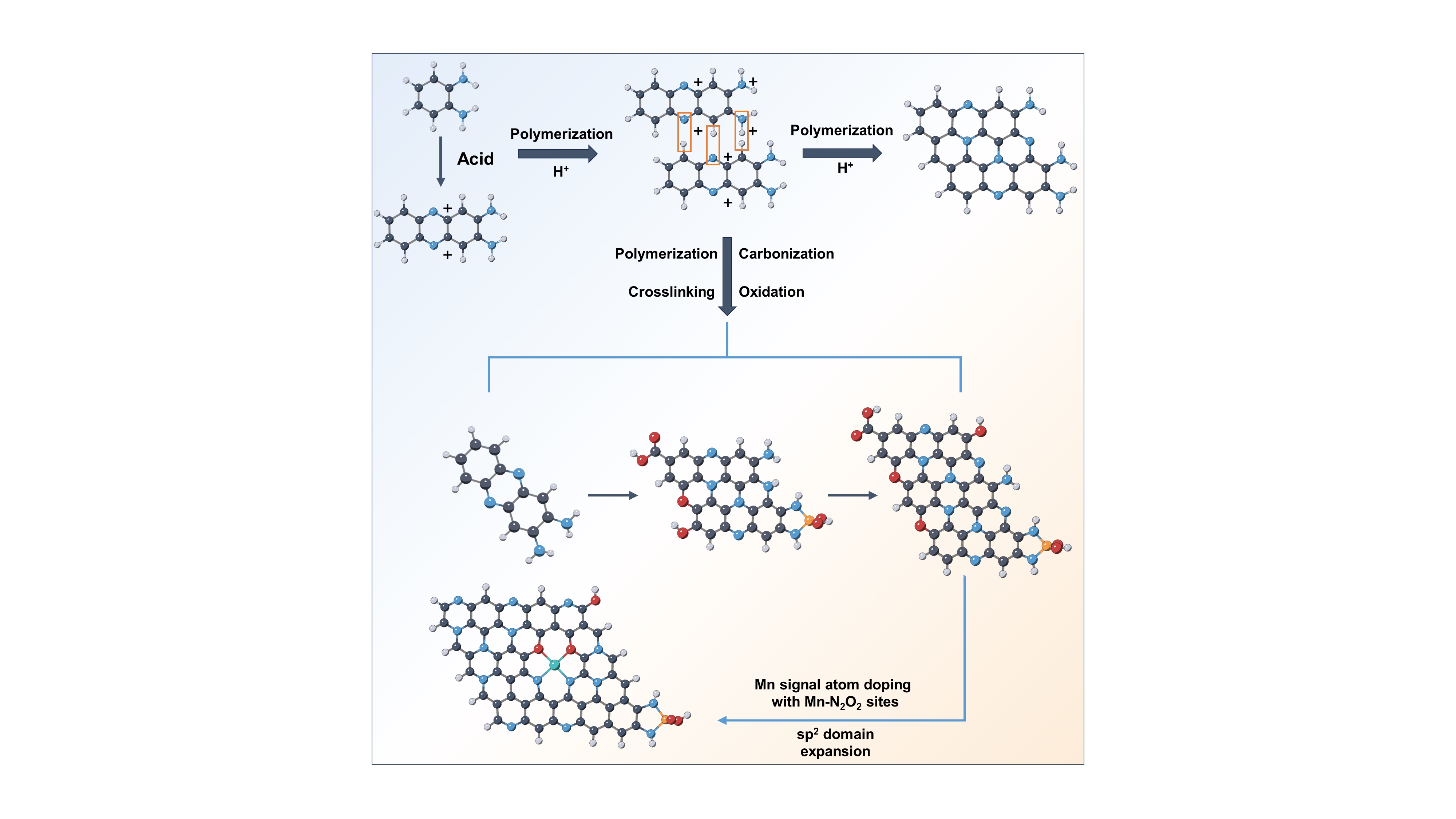 |
| --- |
| **Figure S13.** Schematic diagram of the molecular model construction process for Ⅰ-Y-CDs, Ⅱ-R-CDs, Ⅲ-R-CDs, and Ⅳ-R-Mn-CDs |

| 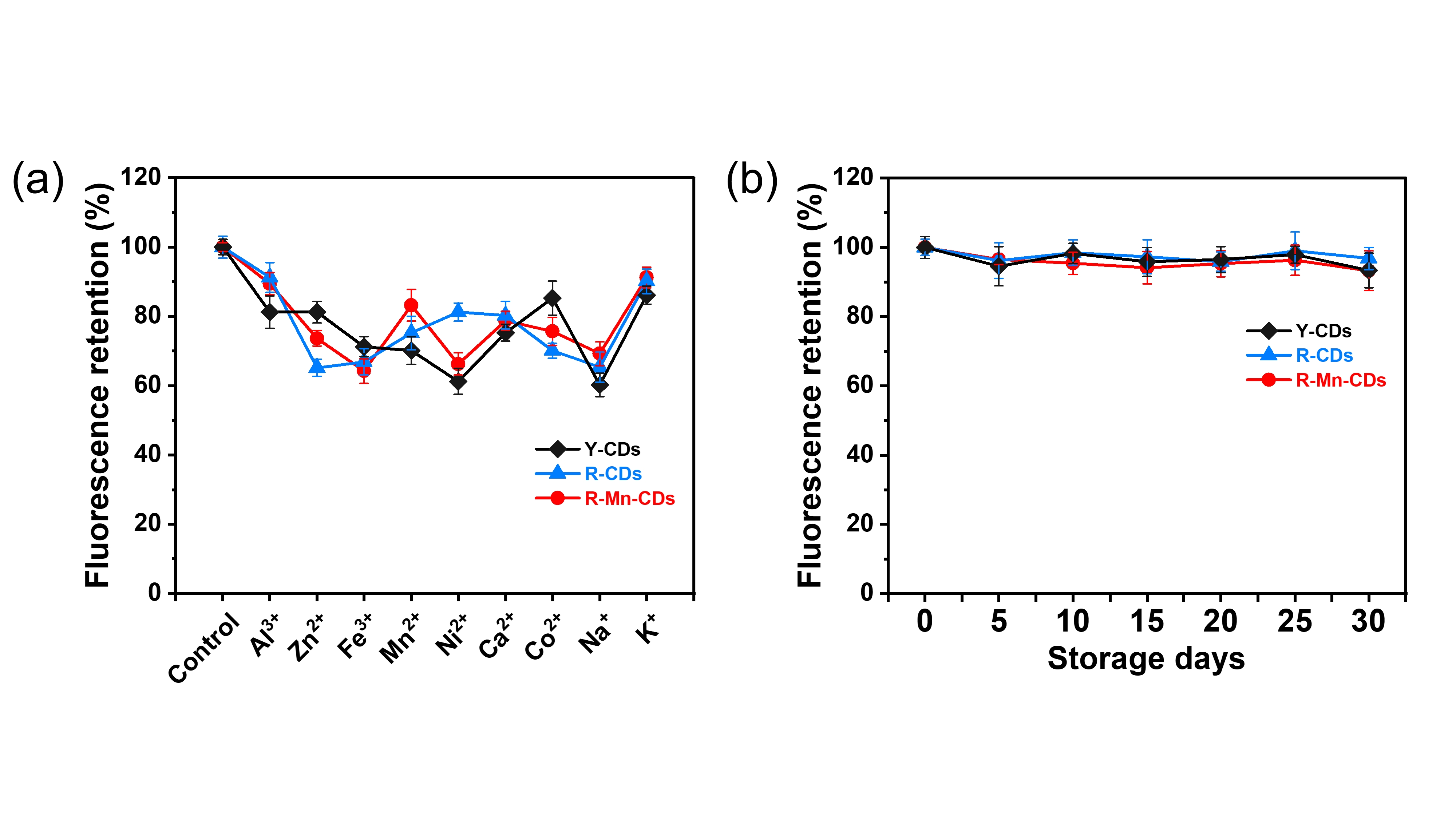 |
| --- |
| **Figure S14.** Fluorescence stability of Y-CDs, R-CDs, and R-Mn-CDs in (a) various metallic salt solutions and (b) during storage for 30 days. |

| 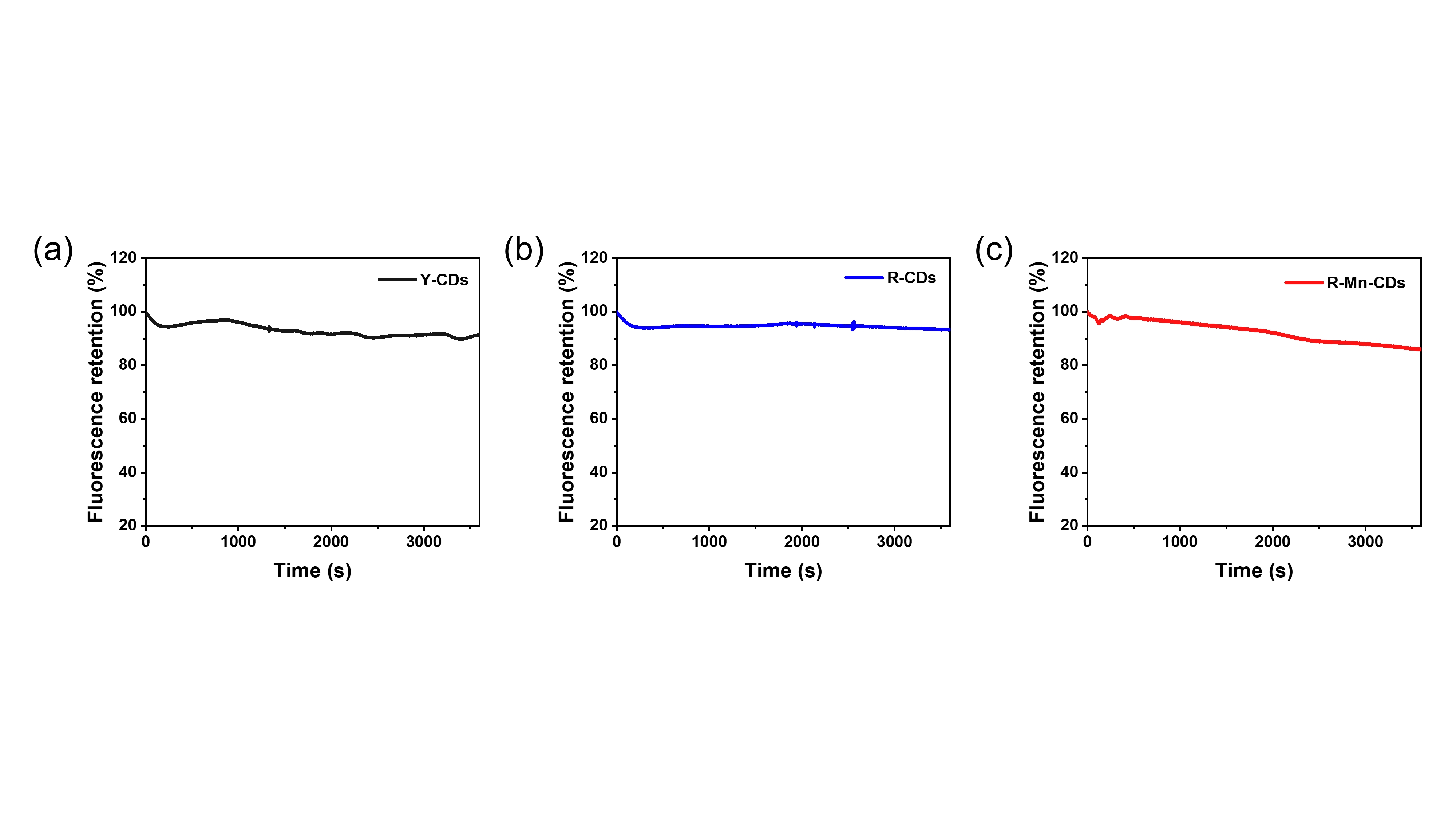 |
| --- |
| **Figure S15.** Fluorescence stability of (a) Y-CDs, (b) R-CDs, and (c) R-Mn-CDs under continuous exposure to excitation light at 420 nm for 60 minutes for Y-CDs, and at 610 nm for 60 minutes for R-CDs and R-Mn-CDs. |

| 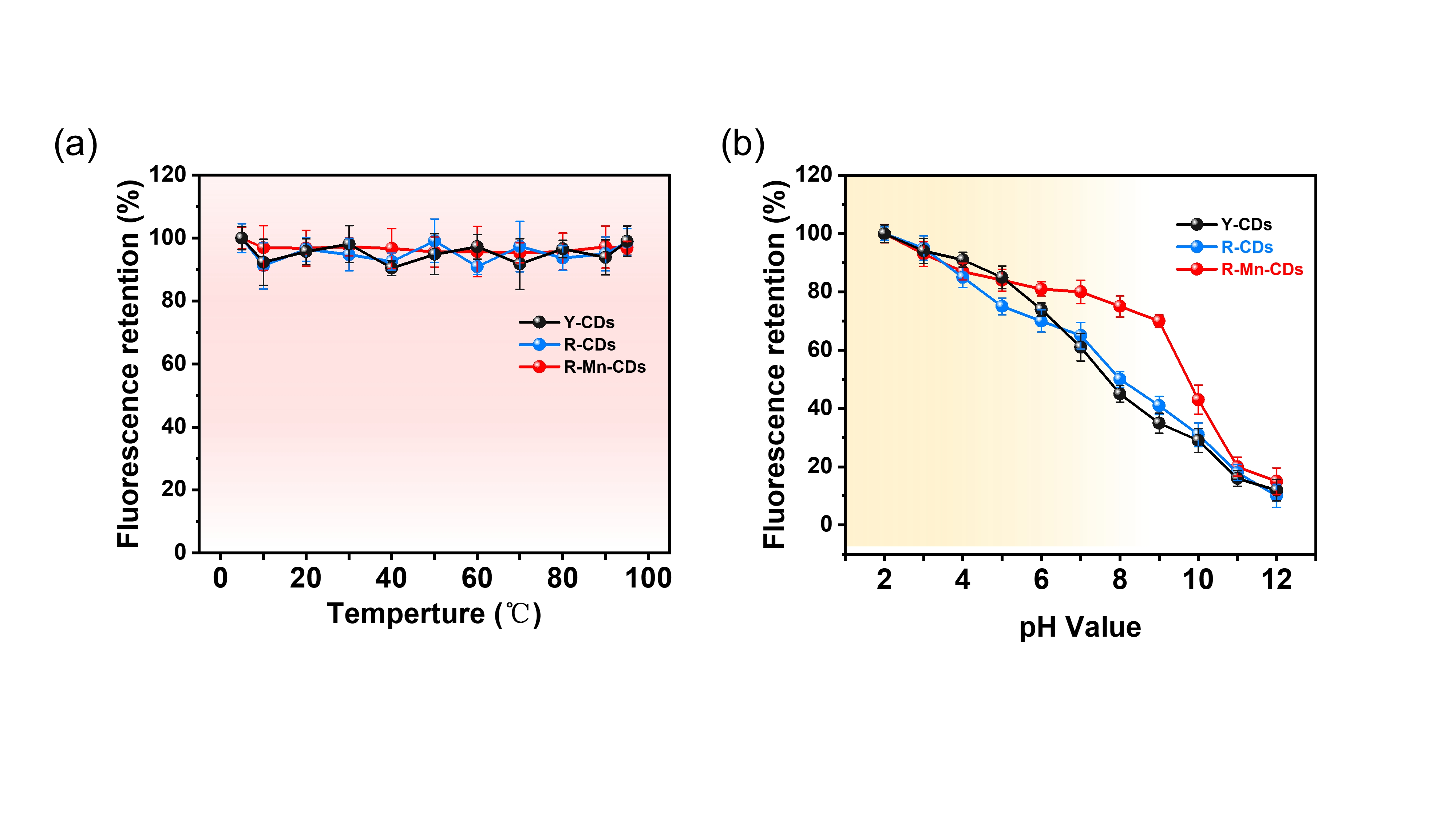 |
| --- |
| **Figure S16.** Fluorescence stability of Y-CDs, R-CDs, and R-Mn-CDs at various (a) temperatures and (b) pH values |

| 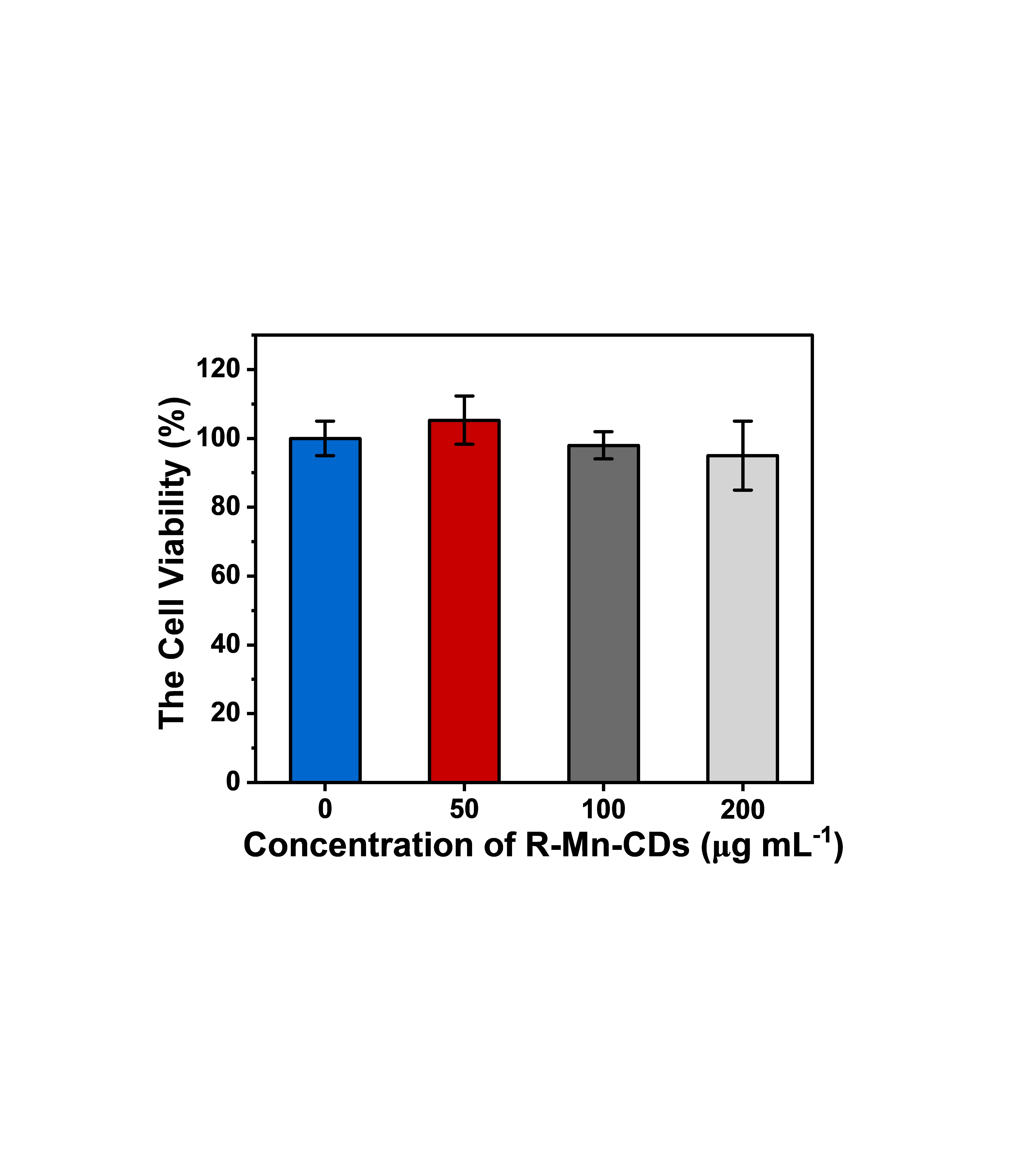 |
| --- |
| **Figure S17.** Survival rate of PC-3 cells in different concentrations of R-Mn-CDs dispersed in PBS buffer solution. |

| 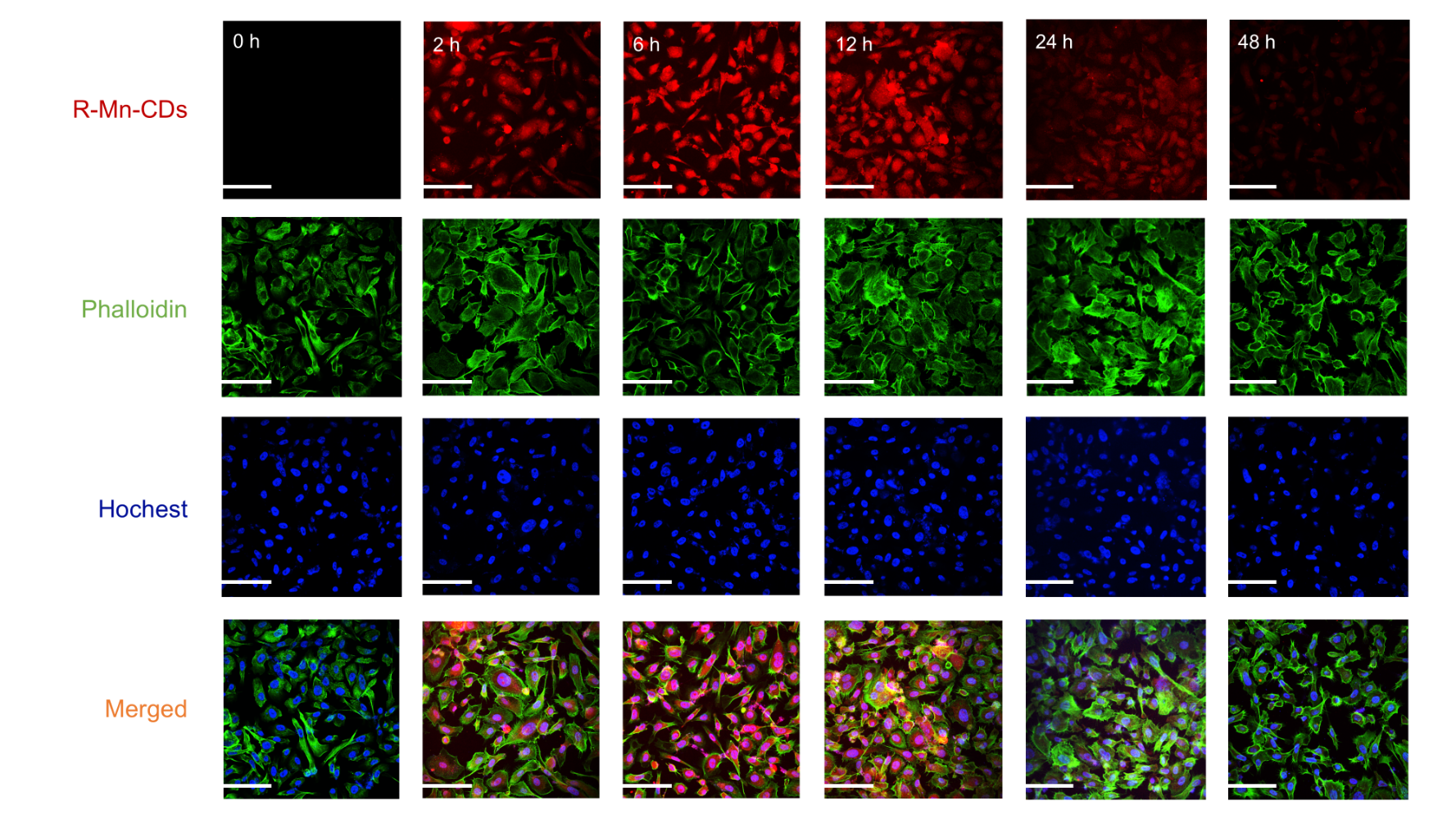 |
| --- |
| **Figure S18.** Confocal fluorescence microscope images of PC-3 cells treated with R-Mn-CDs for different time (0, 2, 6, 12, 24, and 48 h). The red light emission is attributed to R-Mn-CDs, while the green light is produced by gibberellin peptide. In addition, the blue light originates from DPAI. Scale bar = 80 µm |

| 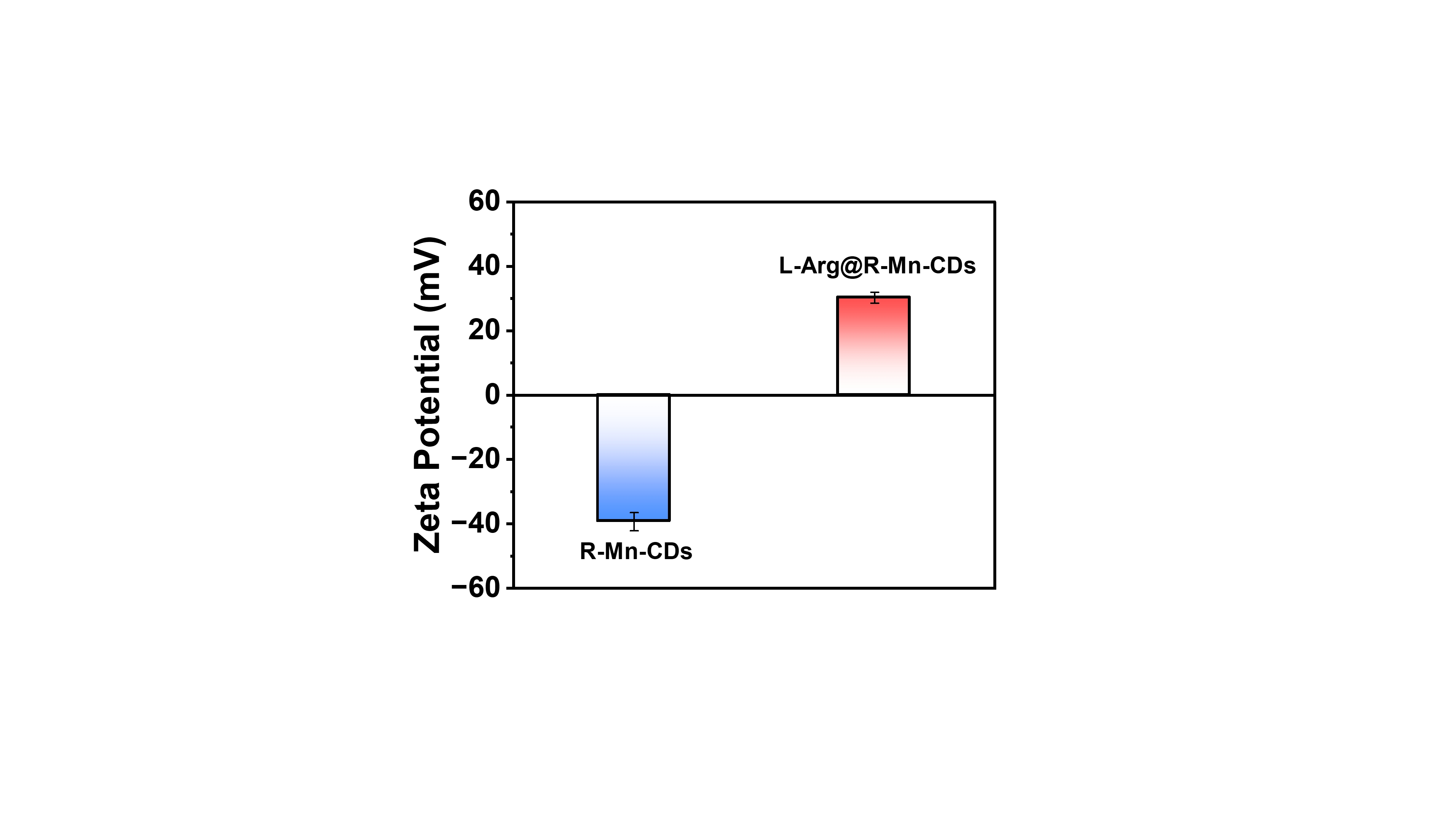 |
| --- |
| **Figure S19.** The Zeta Potential histograms for R-CDs and R-Mn-CDs. |

| 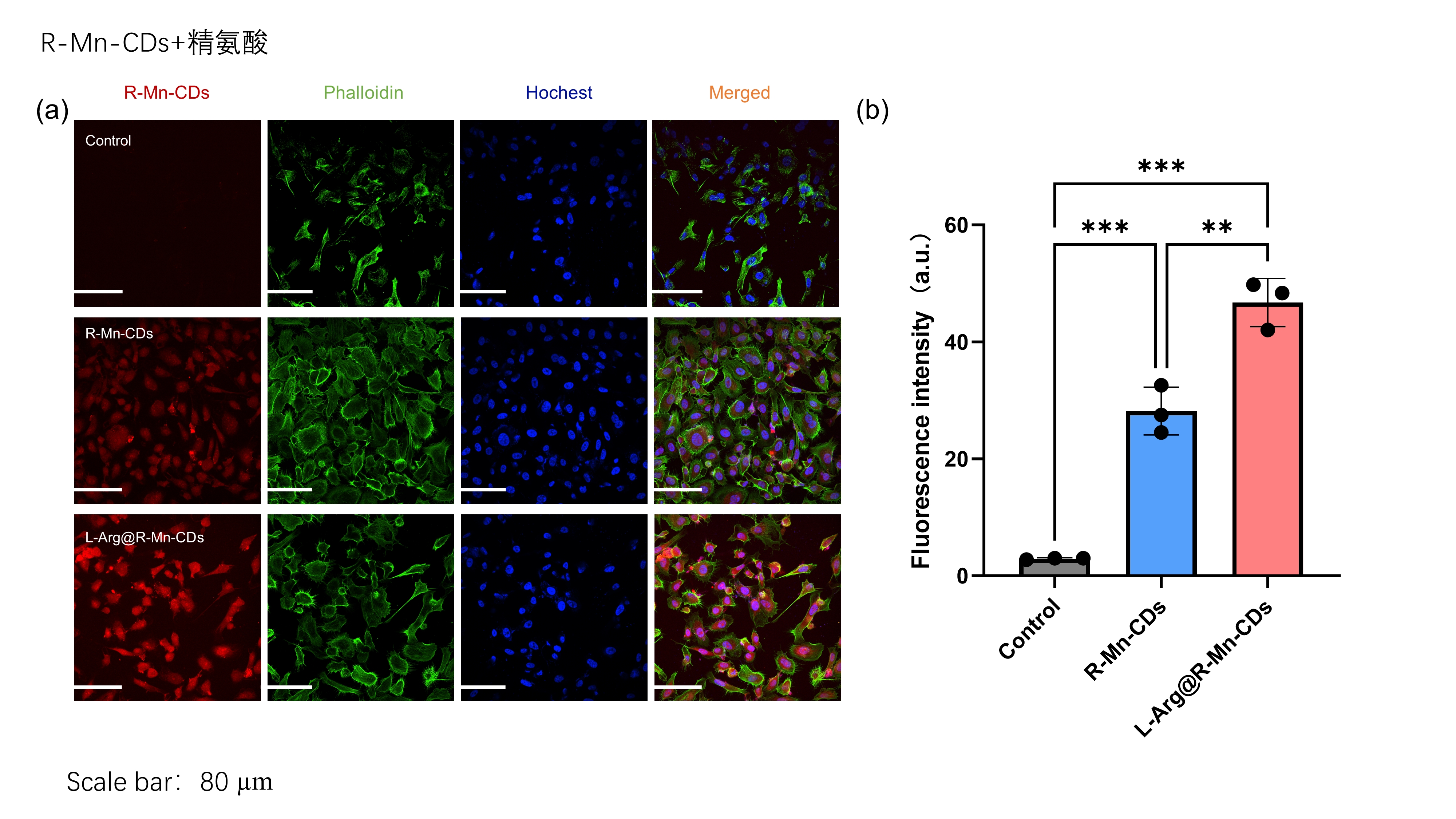 |
| --- |
| **Figure S20.** (a) The confocal fluorescence images of PC-3 cells co-cultured with R-Mn-CDs and L-Arg@R-Mn-CDs for 6 hours, respectively. The control group does not add any materials. The red light emission is attributed to R-Mn-CDs, while the green light is produced by gibberellin peptide. In addition, the blue light originates from DPAI. Scale bar: 80 µm (b) The comparison of the red fluorescence intensity between the control group, R-Mn-CDs group, and L-Arg@R-Mn-CDs group, **p < 0.01, ***p < 0.001 |

| 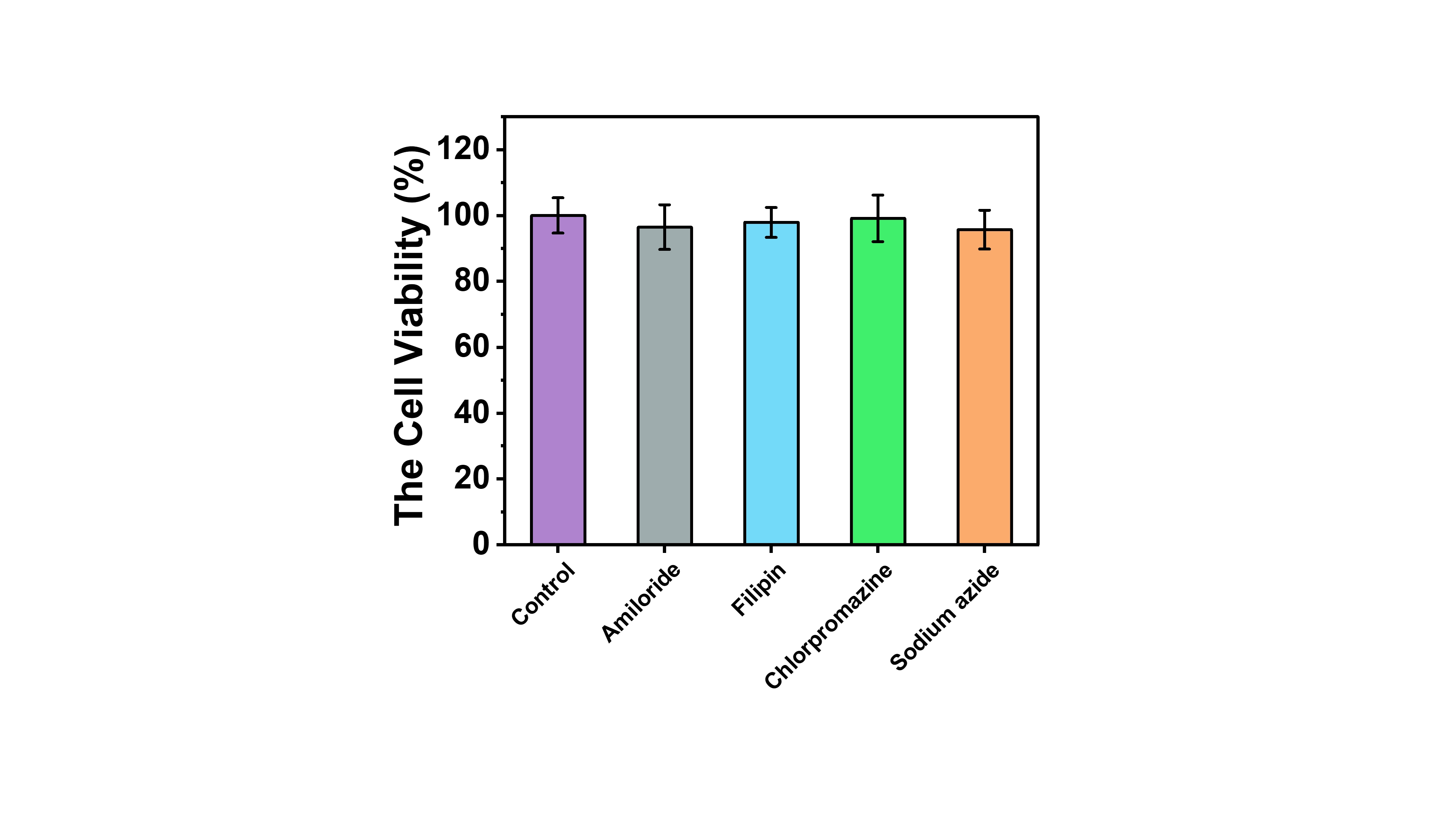 |
| --- |
| **Figure S21.** Cytotoxicity of endocytosis inhibitors in PC-3 cells |

| 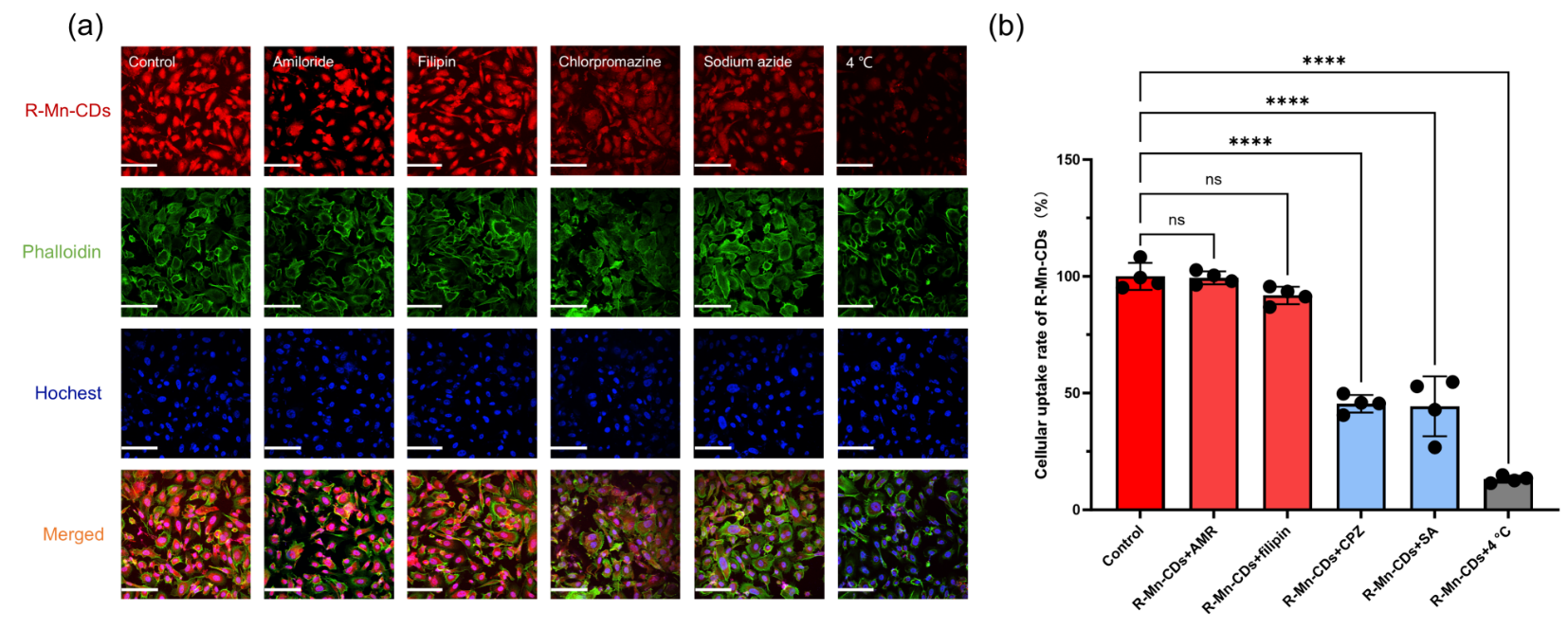 |
| --- |
| **Figure S22**. (a) Confocal microscopic images of the PC-3 cells stained with R-Mn-CDs (100 µg mL^-1^) only (control), treated with R-Mn-CDs (100 μg mL^-1^) and 4 ℃, or one of the endocytosis inhibitors (i.e., amiloride, Filipin, Chlorpromazine, and Sodium azide). Scale bar = 80 µm (b) Comparison of the red fluorescence intensity of these groups, ***p < 0.001. |

| 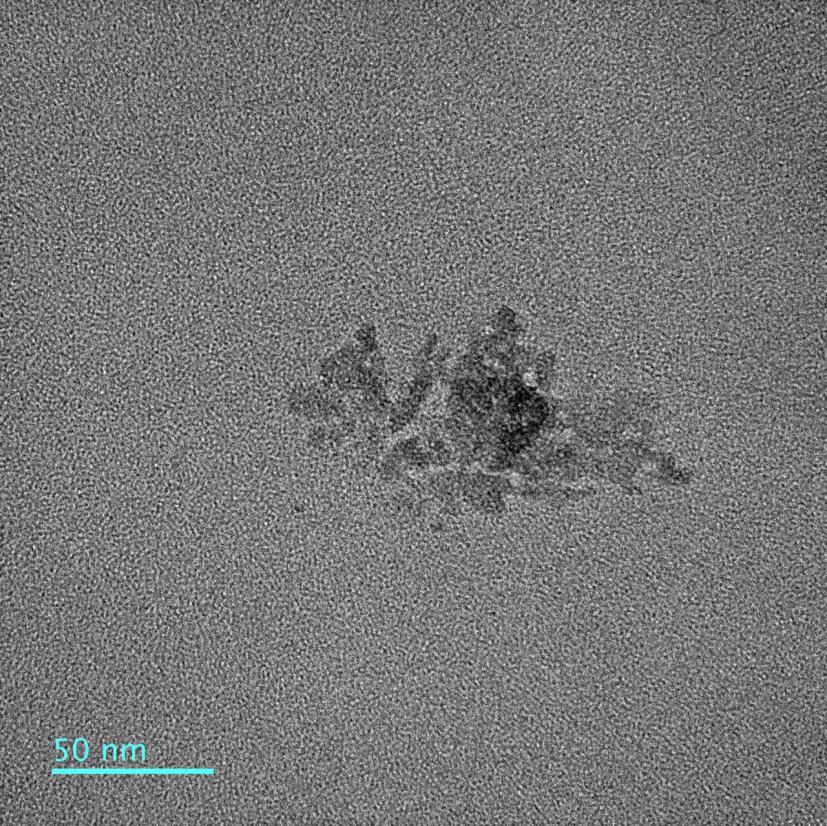 |
| --- |
| **Figure S23.** TEM images of R-Mn-CDs under high concentration (20 mg mL^-1^) |

| 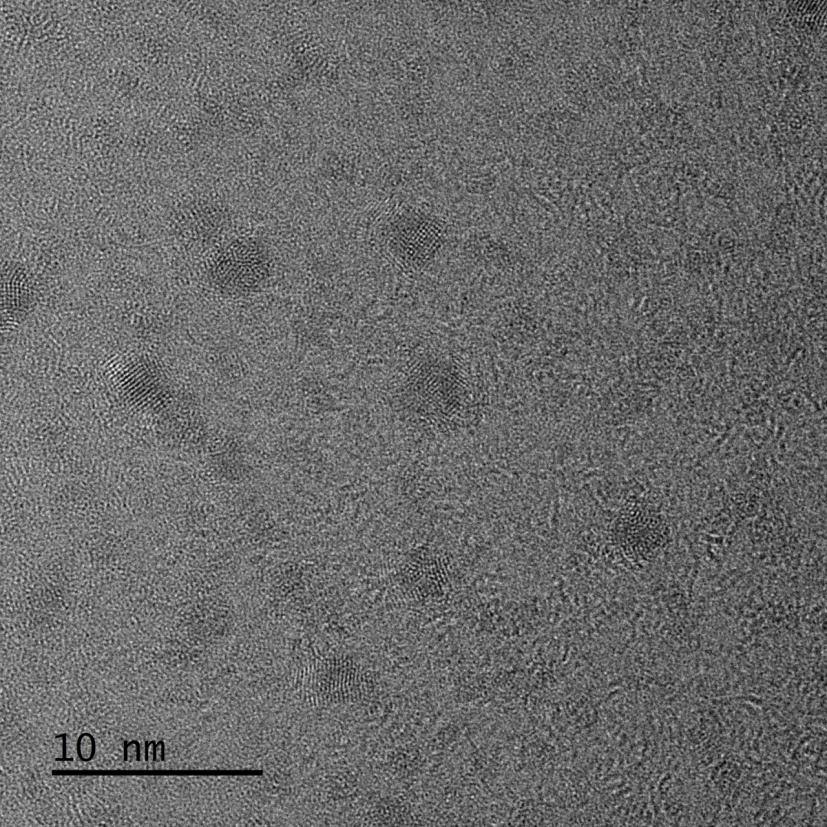 |
| --- |
| **Figure S24.** The TEM images of R-Mn-CDs collected urine at 6 hours post-injection. |

| 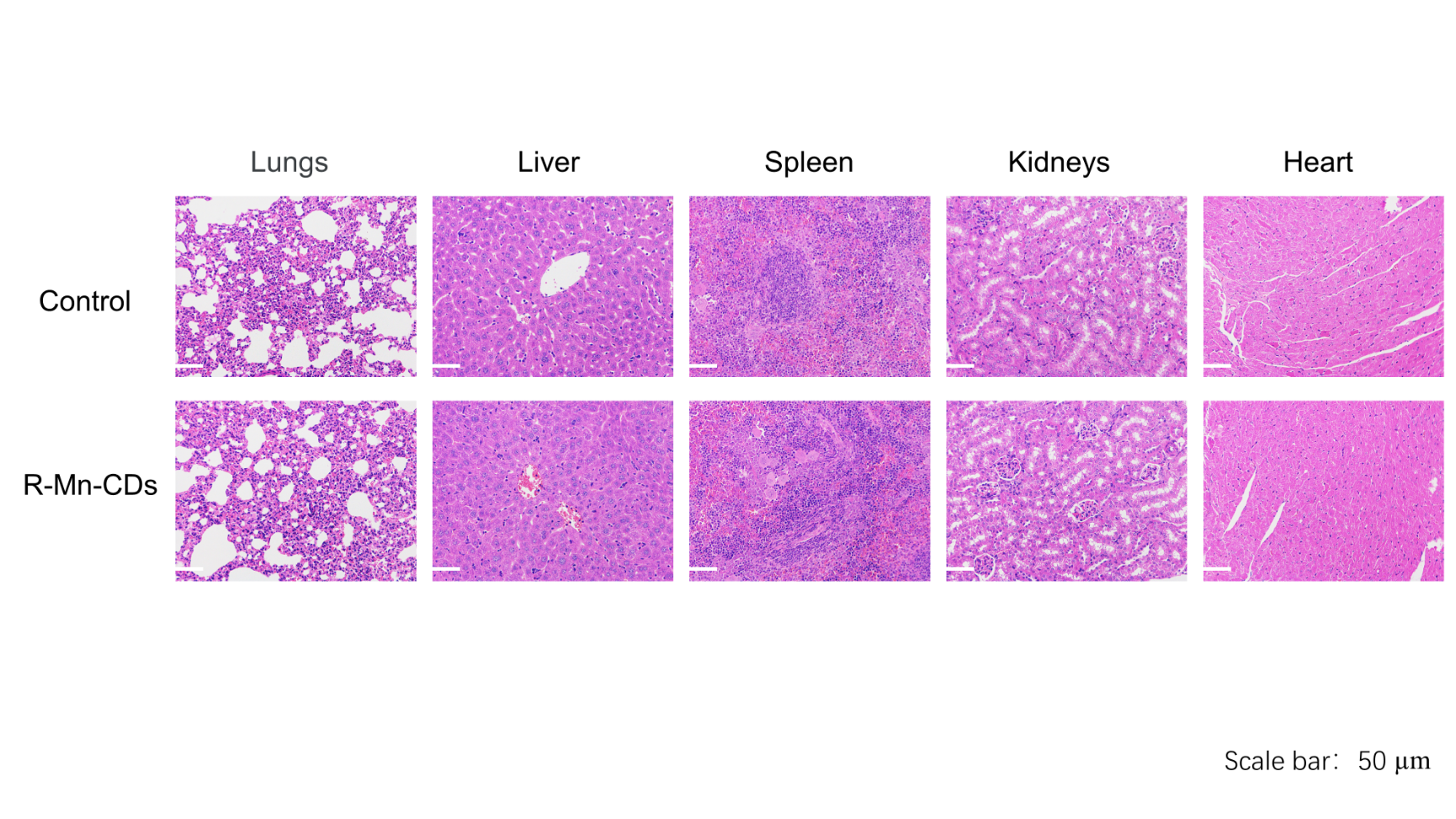 |
| --- |
| **Figure S25.** Histological evaluation of R-Mn-CDs toxicity *in vivo*. Heart, kidney, bladder, liver, lung, and spleen were collected from the control untreated mice and R-Mn-CDs-treated mice via intravenous injection. No symptoms of inflammation and/or lesion were observed in the images. Scale bar = 50 µm |

| 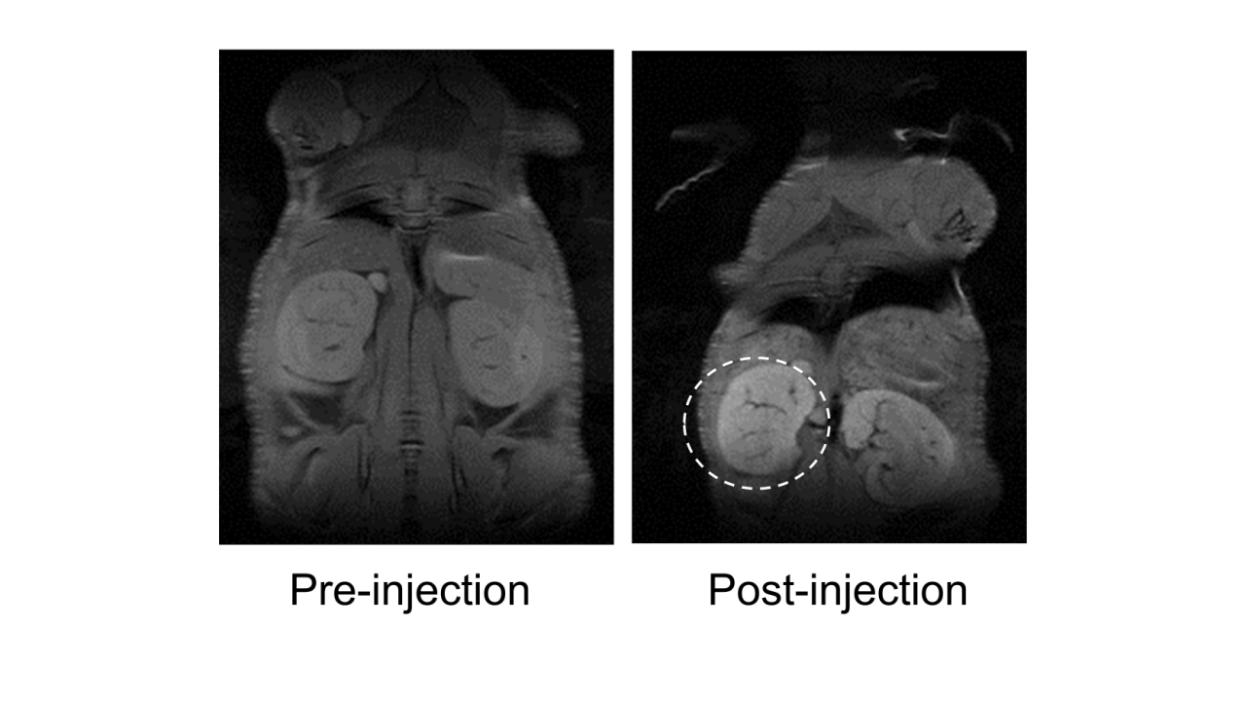 |
| --- |
| **Figure S26.** *In vivo* MRI of mice before and after 1.5 h post-injection of R-Mn-CDs intravenously. |

| 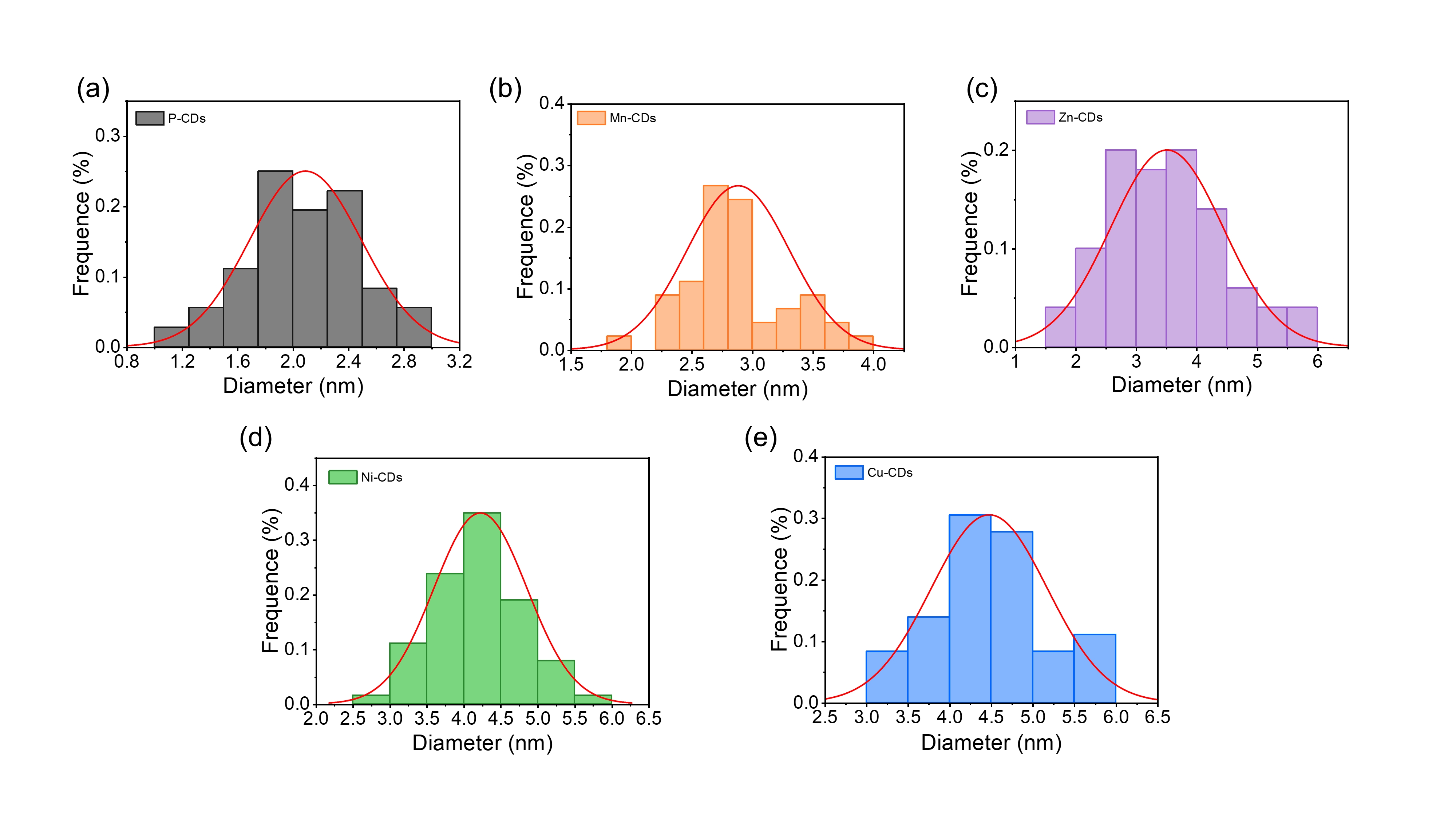 |
| --- |
| **Figure S27.** Size distribution of (a) P-CDs, (b) Mn-CDs, (c) Zn-CDs, (d) Ni-CDs and (e) Cu-CDs. |

**Table S1.** The comparison of FLQYs for R-CDs without surface modification in water prepared using various precursors and synthetic methods.

| **Carbon precursor** | **Preparation method** | **Emission wavelength** | **Fluorescence quantum yield in water** | **References** |
| --- | --- | --- | --- | --- |
| Citric acid, Urea | Solvothermal treatment | 589 nm | 6.0% | [10] |
| Citric acid, Urea, PEI | Solvothermal treatment | 600 nm | 25.0% | [11] |
| Perylene derivatives | Solvothermal treatment | 745 nm | 8.3% | [12] |
| OPD, HNO_3_ | Hydrothermal treatment | 630 nm | 10.8% | [13] |
| OPD, Dopamine, HCl | Hydrothermal treatment | 710 nm | 26.3% | [14] |
| PPD, OPD, Dopamine, HCl | Hydrothermal treatment | 640 nm | 25.5% | [15] |
| Citric acid, Urea | Solvothermal treatment | 628 nm | 18.5% | [16] |
| Citric acid, Tris | Solvothermal treatment | 608 nm | 34.2% | [17] |
| OPD, PA, MnCl_2_ | Hydrothermal method | 625 nm | 41.3% | This work |

**Table S2.** Structural parameters extracted from the Cu K-edge EXAFS fitting. (S_0_^2^=0.81)

| sample | Scattering pair | CN | R(Å) | σ^2^(10^-3^Å^2^) | ΔE_0_(eV) | R factor |
| --- | --- | --- | --- | --- | --- | --- |
| R-Mn-CDs | Mn-O | 2.3 | 2.19 | 4.1 | 1.5 | 0.0016 |
|  | Mn-N | 2.1 | 2.00 | 4.3 | 1.5 |  |

S_0_^2^ is the amplitude reduction factor; CN is the coordination number; R is interatomic distance (the bond length between central atoms and surrounding coordination atoms); σ^2^ is Debye-Waller factor (a measure of thermal and static disorder in absorber-scatterer distances); ΔE_0_ is edge-energy shift (the difference between the zero kinetic energy value of the sample and that of the theoretical model). R factor is used to value the goodness of the fitting.

Error bounds that characterize the structural parameters obtained by EXAFS spectroscopy were estimated as N ± 20%; R ± 1%; σ^2^ ± 20%; ΔE_0_ ± 20%.

**Table S3**. The content of the corresponding metal atoms in different TMAs-doped CDs determined by ICP.

| **Sample Name** | **Tested element** | **Content of the measured element (%)** |
| --- | --- | --- |
| R-Mn-CDs | Mn | 1.46 |
| Mn-CDs | Mn | 1.32 |
| Zn-CDs | Zn | 1.61 |
| Ni-CDs | Ni | 2.15 |
| Cu-CDs | Cu | 2.63 |

**Table S4**. The comparison of the *T_1_* value of R-Mn-CDs to that of other reported nanomaterials.

| **Material Name** | **Elements Responsible for Imaging** | ***T_1_* value (mM^-1^ s^-1^)** | **References** |
| --- | --- | --- | --- |
| Ni-CDs | Ni | 0.52 | [18] |
| Mn-CDs | Mn | 6.97 | [19] |
| MnFe_2_O_4_ | Mn and Fe | 8.01 | [20] |
| MnO_2_/Gd_2_O_3_ | Mn and Ga | 0.339 | [21] |
| Ga-DOTA | Ga | 4.45 | [22] |
| Gadoteric acid | Ga | 3.80 | [23] |
| R-Mn-CDs | Mn | 5.82 | This work |

**Table S5**. The PLQY of different TMAs-doped CDs

| **Material Name** | **Excitation wavelength (nm)** | **Emission wavelength range (nm)** | **PLQY (%)** |
| --- | --- | --- | --- |
| P-CDs | 365 | 385-700 | 4.2 |
| Mn-CDs | 365 | 385-700 | 10.3 |
| Zn-CDs | 370 | 390-700 | 25.7 |
| Ni-CDs | 360 | 380-700 | 36.4 |
| Cu-CDs | 370 | 390-700 | 51.6 |

**Reference**

[1] P. E. Blöchl, *Phys. Rev. B* **1994**, *50*, 17953.

[2] J. P. Perdew, J. A. Chevary, S. H. Vosko, K. A. Jackson, M. R. Pederson, D. J. Singh, C. Fiolhais, *Phys. Rev. B* **1992**, *46*, 6671.

[3] J. P. Perdew, K. Burke, M. Ernzerhof, *Phys. Rev. Lett.* **1996**, *77*, 3865.

[4] S. Kirklin, J. E. Saal, B. Meredig, A. Thompson, J. W. Doak, M. Aykol, S. Rühl, C. Wolverton, *Npj Comput. Mater.* **2015**, *1*, 1.

[5] S. Grimme, J. Antony, S. Ehrlich, H. Krieg, *J. Chem. Phys.* **2010**, *132*, 154104.

[6] M. Dolg, U. Wedig, H. Stoll, H. Preuss, *J. Chem. Phys.* **1987**, *86*, 866.

[7] D. Andrae, U. Häußermann, M. Dolg, H. Stoll, H. Preuß, *Theor. Chim. Acta* **1990**, *77*, 123.

[8] P. C. Hariharan, J. A. Pople, *Theor. Chim. Acta* **1973**, *28*, 213.

[9] W. J. Hehre, R. Ditchfield, J. A. Pople, *J. Chem. Phys.* **1972**, *56*, 2257.

[10] H. Zhang, G. Wang, Z. Zhang, J. H. Lei, T.-M. Liu, G. Xing, C.-X. Deng, Z. Tang, S. Qu, *Light Sci. Appl.* **2022**, *11*, 113.

[11] L. Wang, B. Wang, E. Liu, Y. Zhao, B. He, C. Wang, G. Xing, Z. Tang, Y. Zhou, S. Qu, *Chin. Chem. Lett.* **2022**, *33*, 4111.

[12] Y. Liu, J. H. Lei, G. Wang, Z. Zhang, J. Wu, B. Zhang, H. Zhang, E. Liu, L. Wang, T.-M. Liu, G. Xing, D. Ouyang, C.-X. Deng, Z. Tang, S. Qu, *Adv. Sci.* **2022**, *9*, 2202283.

[13] J. Liu, D. Li, K. Zhang, M. Yang, H. Sun, B. Yang, *Small* **2018**, *14*, 1703919.

[14] S. Lu, L. Sui, J. Liu, S. Zhu, A. Chen, M. Jin, B. Yang, *Adv. Mater.* **2017**, *29*, 1603443.

[15] X. Ye, Y. Xiang, Q. Wang, Z. Li, Z. Liu, *Small* **2019**, *15*, 1901673.

[16] H. Zhang, Q. Li, S. Wang, X. Yu, B. Wang, G. Chen, L. Ren, J. Li, M. Jin, J. Yu, *Nano Res.* **2023**, *16*, 4170.

[17] Y. Zhang, H. Song, L. Wang, J. Yu, B. Wang, Y. Hu, S.-Q. Zang, B. Yang, S. Lu, *Angew. Chem. Int. Ed.* **2021**, *60*, 25514.

[18] B. Tian, S. Liu, L. Feng, S. Liu, S. Gai, Y. Dai, L. Xie, B. Liu, P. Yang, Y. Zhao, *Adv. Funct. Mater.* **2021**, *31*, 2100549.

[19] Q. Jia, J. Ge, W. Liu, X. Zheng, S. Chen, Y. Wen, H. Zhang, P. Wang, *Adv. Mater.* **2018**, *30*, 1706090.

[20] H. Zhang, Y. Guo, J. Jiao, Y. Qiu, Y. Miao, Y. He, Z. Li, C. Xia, L. Li, J. Cai, K. Xu, X. Liu, C. Zhang, B.-H. Bay, S. Song, Y. Yang, M. Peng, Y. Wang, H. Fan, *Nat. Biomed. Eng.* **2023**, *7*, 221.

[21] F. Wang, L. Wen, J. Liu, W. Peng, Z. Meng, Q. Chen, Y. Wang, B. Ke, Y. Guo, P. Mi, *Biomaterials* **2020**, *230*, 119614.

[22] H.-L. Wang, D. Liu, J.-H. Jia, J.-L. Liu, Z.-Y. Ruan, W. Deng, S. Yang, S.-G. Wu, M.-L. Tong, *Natl. Sci. Rev.* **2023**, *10*, nwad036.

[23] R. K. Kawassaki, M. Romano, M. Klimuk Uchiyama, R. M. Cardoso, M. S. Baptista, S. H. P. Farsky, K. T. Chaim, R. R. Guimarães, K. Araki, *Nano Lett.* **2023**, *23*, 5497.
